# Armored kinorhynch-like scalidophoran animals from the early Cambrian

**DOI:** 10.1038/srep16521

**Published:** 2015-11-26

**Authors:** Huaqiao Zhang, Shuhai Xiao, Yunhuan Liu, Xunlai Yuan, Bin Wan, A. D. Muscente, Tiequan Shao, Hao Gong, Guohua Cao

**Affiliations:** 1 Key Laboratory of Economic Stratigraphy and Palaeogeography, Chinese Academy of Sciences (Nanjing Institute of Geology and Palaeontology), Nanjing 210008, China; 2 Department of Geosciences, Virginia Tech, Blacksburg, Virginia 24061, USA; 3 College of Earth Science and Resources, Chang’an University, Xi’an 710054, China; 4 State Key Laboratory of Palaeobiology and Stratigraphy, Nanjing Institute of Geology and Palaeontology, Chinese Academy of Sciences, Nanjing 210008, China; 5 Virginia Tech – Wake Forest University School of Biomedical Engineering and Sciences, Virginia Tech, Blacksburg, VA 24061, USA

## Abstract

Morphology-based phylogenetic analyses support the monophyly of the Scalidophora (Kinorhyncha, Loricifera, Priapulida) and Nematoida (Nematoda, Nematomorpha), together constituting the monophyletic Cycloneuralia that is the sister group of the Panarthropoda. Kinorhynchs are unique among living cycloneuralians in having a segmented body with repeated cuticular plates, longitudinal muscles, dorsoventral muscles, and ganglia. Molecular clock estimates suggest that kinorhynchs may have diverged in the Ediacaran Period. Remarkably, no kinorhynch fossils have been discovered, in sharp contrast to priapulids and loriciferans that are represented by numerous Cambrian fossils. Here we describe several early Cambrian (~535 million years old) kinorhynch-like fossils, including the new species *Eokinorhynchus rarus* and two unnamed but related forms. *E. rarus* has characteristic scalidophoran features, including an introvert with pentaradially arranged hollow scalids. Its trunk bears at least 20 annuli each consisting of numerous small rectangular plates, and is armored with five pairs of large and bilaterally placed sclerites. Its trunk annuli are reminiscent of the epidermis segments of kinorhynchs. A phylogenetic analysis resolves *E. rarus* as a stem-group kinorhynch. Thus, the fossil record confirms that all three scalidophoran phyla diverged no later than the Cambrian Period.

As a small ecdysozoan phylum, the Kinorhyncha includes ~240 extant species of exclusively marine, holobenthic, free-living, meiofaunal animals^1^. The body of kinorhynchs is divided into a head including a protrusible mouth cone with circlets of pentaradially arranged teeth and an eversible introvert with circlets of pentaradially arranged scalids, a neck, and a trunk with 11 segments, also known as zonites or macroannuli^1^,^2^. Kinorhynchs are exceptions among cycloneuralians in that their epidermis is divided into a small number of macroannuli consisting of cuticular plates^1^, whereas other cycloneuralians do not have epidermis segmentation or their epidermis is divided into a large number of narrow annulations termed microannuli (e.g., priapulids)^2^. In addition, several of their organ systems including circular muscles, longitudinal muscles, and ganglions are also segmented^2^,^3^,^4^. As such, kinorhynchs offer an excellent model to investigate the origin(s) of body segmentation, provided that their exact phylogenetic position is confidently resolved and their fossil record is adequately preserved.

However, the exact phylogenetic relationship among ecdysozoan phyla remains unresolved. Morphology-based phylogenetic analyses support the monophyly of scalidophorans (kinorhynchs, loriciferans, and priapulids) and nematoids (nematods, nematomorphs), together constituting the cycloneuralians which are a sister group of panarthropods^3^,^5^,^6^,^7^,^8^,^9^. On the other hand, some molecular phylogenetic analyses question the monophyly of cycloneuralians and propose that the nematoids are more closely related to panarthropods than to scalidophorans^10^,^11^,^12^, although others do support a monophyletic cycloneuralian clade^13^. Similarly, the monophyly of scalidophorans has also been challenged by molecular phylogenetic analyses that exclude loriciferans from the scalidophorans and instead favor a loriciferan-nematomorph^11^ or a loriciferan-panarthropod relationship^14^. Within the traditionally recognized scalidophorans (kinorhynchs, loriciferans, and priapulids), the three possible sister-group relationships—kinorhynch-loriciferan^3^, loriciferan-priapulid^5^,^7^, and kinorhynch-priapulid^11^—have all been proposed.

On the paleontological side, the fossil record of scalidophoran phyla is highly uneven. Abundant priapulid-like fossils (most representing stem-group scalidophorans or stem-group priapulids)^15^,^16^,^17^,^18^,^19^ and several loriciferan-like fossils^7^,^8^,^9^ have been reported from the Cambrian Period, but thus far no kinorhynch fossils have been discovered^1^. The lack of kinorhynch fossils is a prominent knowledge gap, given that molecular clocks suggest an Ediacaran divergence of the scalidophorans^10^ and that Cambrian priapulid-like and loriciferan-like fossils imply the presence of total-group kinorhynchs in Cambrian or older rocks if kinorhynchs are a sister group to loriciferans^3^ or priapulids^11^. Considering that all scalidophorans have a cuticle-bearing but non-biomineralized body, in principle they should have similar potential to be preserved in the fossil record. However, because kinorhynchs are exclusively meiofaunal, there may be a bias toward preservation through phosphatization that is known to selectively preserve millimeter-sized organisms^20^,^21^, as opposed to Burgess Shale-type preservation that accounts for the fossilization of all macrofaunal scalidophorans^7^,^9^,^15^,^16^. Indeed, recent exploration of the phosphatization taphonomic window has revealed numerous microscopic scalidophoran fossils^8^,^17^,^18^,^19^, highlighting the potential of this taphonomic window in filling the gap of kinorhynch fossils.

Here we describe several three-dimensionally phosphatized fossils, including *Eokinorhynchus rarus* gen. et sp. nov. and two unnamed forms, from early Cambrian (~535 Ma) limestones dated to the Fortunian *Anabarites trisulcatus*–*Protohertzina anabarica* Assemblage Zone at Xinli and Xixiang sections, South China (Supplementary Fig. S1)^22^,^23^,^24^. These fossils, particularly *E. rarus*, have characteristic scalidophoran features and show some similarities with (and also key differences from) modern kinorhynchs. The new fossils have the potential to illuminate the Cambrian evolution of scalidophorans and kinorhynchs.

## Results

Scalidophora Lemburg, 1995

*Eokinorhynchus* gen. nov.

### Etymology

*Eo-*, dawn; *kinorhynchus*, kinorhynchs.

### Type species

*Eokinorhynchus rarus* gen. et sp. nov.

### Diagnosis

Same as type species by monotypy.

*Eokinorhynchus rarus* gen. et sp. nov.(

**Figure 1:**
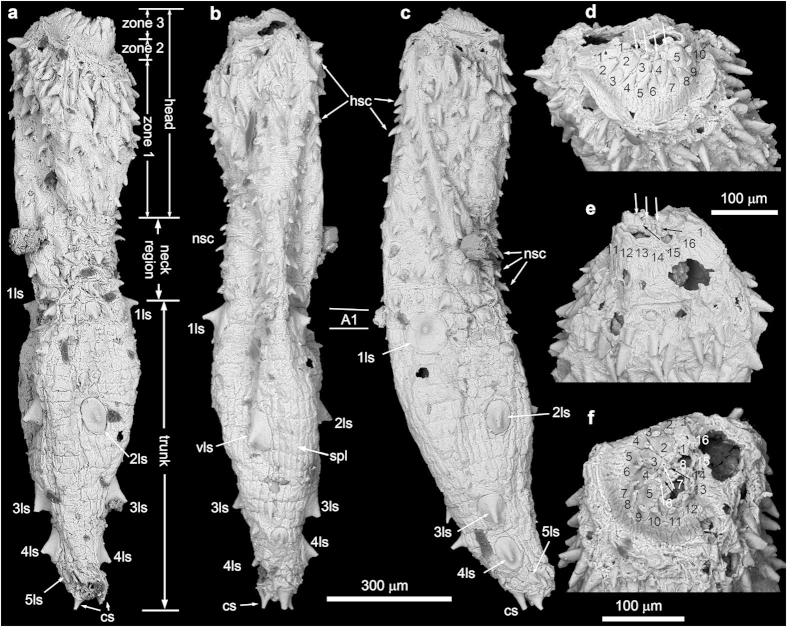
SEM images of *Eokinorhynchus rarus* gen. et sp. nov., holotype, NIGP160400. (**a–c**) Dorsal, ventral, and left lateral views, respectively. (**d–f**) Close-up views of zones 2 and 3 to show the arrangement pattern of pharyngeal teeth (numbered in the two basal circlets), with white arrows denoting the 3rd and black arrows denoting the 4th circlet of pharyngeal teeth. Abbreviations in this and other figures: A1–A20, 1st to 20th trunk annulus; an, anus; cs, caudal spine; hsc, head scalid; ls, large sclerite; 1ls–5ls, 1st to 5th pair of large sclerites; mg, midgut; nsc, neck scalid; spl, small plate; ss, small spine; vls, ventral large sclerite. Scale bar beneath (**c**) applies to (**a–c**), and scale bar beneath (**d**) applies to (**d–e**). SEM images acquired by authors.

**Figure 2:**
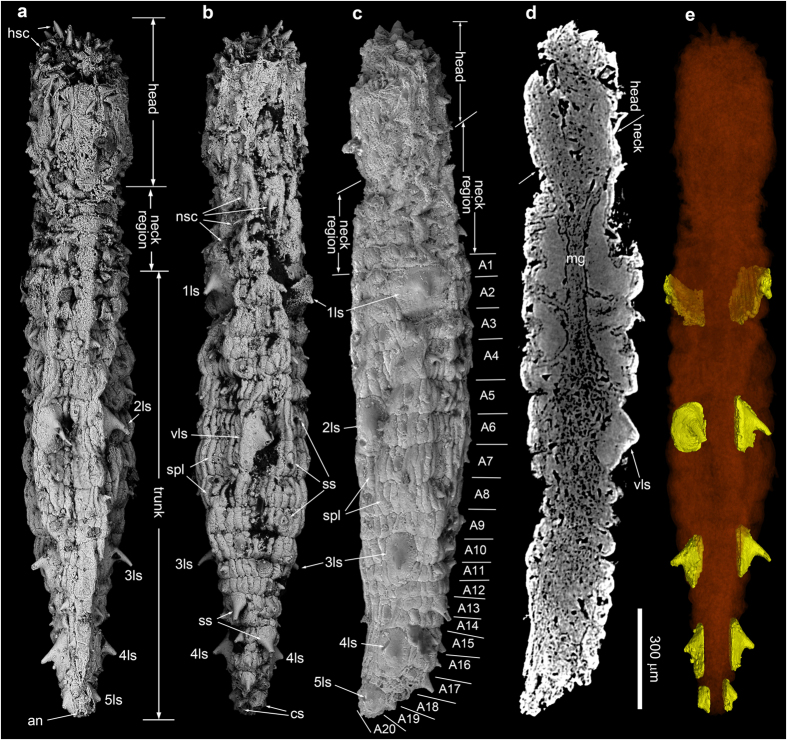
SEM and microCT images of *Eokinorhynchus rarus* gen. et sp. nov., paratype, NIGP160401. (**a–c**) SEM images of dorsal, ventral, and right lateral views respectively. (**d**) microCT saggital section, with dorsal side to the left. (**e**) Dorsal view of microCT reconstruction, with large sclerites rendered yellow and everything else semi-transparent to show their bilateral arrangement. Scale bar applies to all images. SEM and microCT images acquired by authors.

**Figure 3:**
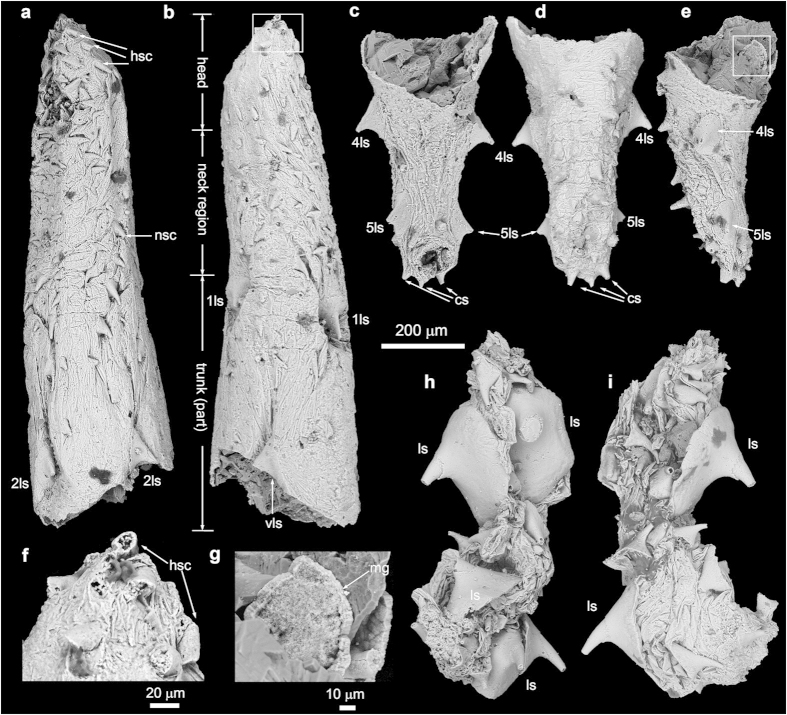
SEM images of *Eokinorhynchus rarus* gen. et sp. nov., paratype (NIGP160402) and a fragmented trunk (NIGP160414). NIGP160402 was originally a complete specimen, but was accidentally broken during preparation, and only the head (NIGP160402a) and tail (NIGP160402b) were recovered and illustrated. (**a,b**) Dorsal and ventral views of NIGP160402a, with white rectangle marking area magnified in (**f**). (**c–e**) Dorsal, ventral, and left lateral views of NIGP160402b, with white rectangle marking area magnified in (**g**). (**h,i**) Two opposite views of NIGP160414. Scale bar beneath (**c**) applies to (**a–e**,**h,i**). SEM images acquired by authors.

Figures 1, 2, 3; Supplementary S2–S3; Supplementary Table S1; Supplementary Movies S1–S4)

### Etymology

From Latin, *rarus*, rare.

### Type specimens

Holotype NIGP160400 (Fig. 1). Paratypes NIGP160401 (Fig. 2) and NIGP160402 (Fig. 3a–g). Deposited at Nanjing Institute of Geology and Palaeontology (NIGP).

### Additional material

NIGP160414 (Fig. 3h,i).

### Locality and horizon

Xinli section^25^, Nanjiang County, Sichuan Province (Supplementary Fig. S1); Xinli Member, Dengying Formation, small shelly fossils *Anabarites trisulcatus*–*Protohertzina anabarica* Assemblage Zone^23^, about 535 Ma, Fortunian Stage^22^.

### Diagnosis

Worm-like animal composed of a head, a neck region, and a trunk. Head consists of an introvert with pentaradially arranged hollow scalids and a pharynx with octaradially arranged teeth. Neck region covered with 5 circlets of neck scalids. Trunk has at least 20 annuli and each annulus is covered with a circlet of tightly sutured small plates and armored with spinose sclerites. Five pairs of large spinose sclerites are bilaterally arranged and a single large spinose sclerite is midventrally located. Two pairs of caudal spines are located slightly ventral to the terminal anus.

### Description

The head is demarcated from the neck by a constriction (Figs 1a and 2a,c,d). It includes a pharynx armed with teeth (zone 3), an unarmed transitional zone (zone 2), and an introvert with head scalids (zone 1). These zones are easily recognizable in the holotype (Fig. 1a), but only zone 1 is partially visible in the paratypes because their pharynx and part of the introvert are retracted (Figs 2a and 3a).

The everted pharynx (zone 3) has at least 4 circlets of radially arranged and anteriorly directed pharyngeal teeth, surrounding a terminal mouth. The outermost circlet has 16 conical teeth (Fig. 1f), and the second circlet has 8 much larger teeth, interdigitally positioned between every pair of teeth in the outermost circlet (Fig. 1d). Two additional circlets are inferred on the basis of partly preserved and much smaller teeth close to the center (Fig. 1d–f).

Zone 2 is unarmed, but has densely-spaced longitudinal wrinkles (Fig. 1d–f). The everted introvert (zone 1) bears 7 circlets of posteriorly directed head scalids (Fig. 1a–c; Supplementary Fig. S2). The first (anteriormost) and second circlets each have 25 radially arranged scalids that are aligned to form 25 longitudinal rows. The following 5 circlets each have 13, 12, 12, 11, and 9 scalids respectively, but they are more irregularly distributed, and do not follow the longitudinal rows defined by the first two circlets. The introvert of the paratypes is only partly everted, thus the arrangement pattern of the scalids is difficult to discern (Figs 2a and 3a). The scalids are conical in shape, internally hollow (Fig. 3f; Supplementary Fig. S3a), and their length decreases posteriorly (Fig. 1a).

The neck region consists of five circlets of neck scalids, which do not form longitudinal rows (Supplementary Fig. S2). Neck scalids are numbered 10, 11, 11, 13, and 12 from the first (anteriormost) to the fifth circlets. They are short and have a slightly expanded base, different from the relatively long and conical introvert scalids.

The trunk begins at the first annulus bearing a pair of large spinose sclerites. The trunk annuli are undifferentiated or poorly differentiated in NIGP160400 and NIGP160402 (Figs 1 and 3a,b), but in NIGP160401 20 trunk annuli are clearly recognizable (“A1–A20” in Fig. 2c). They vary in length, with A4 being the longest (110 μm). They are each covered with a single circlet of ~20–40 tightly sutured rectangular small plates. The trunk is armored with internally hollow small spines (“ss” in Fig. 2b; Supplementary Fig. S3e), which are of various sizes and somewhat irregularly distributed but are more concentrated postero-ventrally (Figs 1b, 2b, 3d; Supplementary Fig. S3e). Some small spines—for example, the two dorsal spines (Fig. 1a) and one ventral spine (Fig. 1b) in A1 of NIGP160400—resemble the neck scalids in size and shape. The five pairs of large spinose sclerites (“1ls–5ls” in Figs 1a–c and 2a–c) distinguish the trunk from the neck, and their arrangement gives the animal a strong bilaterality and dorsoventrality (Fig. 2e; Supplementary Movie S1). They are located ventrolaterally on A1–2, laterodorsally on A5–6, midlaterally on A10–11, midlaterally on A15–17, and laterodorsally on A19–20. In addition, a single large sclerite is placed midventrally on A6–7. These sclerites have an enlarged base straddling two or more annuli and supporting a robust conical spine. A possible anus is present at the posterior end of the trunk (“an” in Fig. 2a; Supplementary Fig. S3b). There are two pairs of caudal spines (“cs” in Figs 1a–c, 2b, 3c,d; Supplementary Fig. S3e) located slightly ventrally to the presumed anus.

The trunk of NIGP160401 is internally filled with diagenetic phosphatic minerals (Supplementary Fig. S3f–i; Supplementary Movies S2–4). The only preserved internal organ is a tubular lumen in the posterior neck and anterior trunk (“mg” in Fig. 2d). It is nearly constant in diameter (~60 μm), widens both anteriorly and posteriorly, has a triangular or oval cross section (Supplementary Fig. S3i; Supplementary Movie S4), and extends to A5. This tubular lumen is interpreted as part of the midgut which is anteriorly connected with the esophagus. The midgut may also be partially preserved in NIGP160402 (Fig. 3e,g).

NIGP160402 (Fig. 3a), NIGP160400 (Fig. 1), and NIGP160401 (Fig. 2) probably represent progressively advanced developmental stages: as the body lengthens, large sclerites enlarge and trunk annuli become better differentiated. A trunk fragment (Fig. 3h), whose large sclerites reach ~290 μm in diameter, may represent a still later developmental stage. If true, the three type specimens may be juveniles or young adults, and mature specimens may have ≥20 trunk annuli.

### Nomenclature note

This article is published in an electronic journal with an ISSN (2045–2322), and has been archived in PubMed Central. Taxonomic nomenclature published in this article conforms to the requirements of the amended International Code of Zoological Nomenclature (ICZN), and hence is available under ICZN. This publication and the nomenclatural acts it contains have been registered in ZooBank (www.zoobank.org). The ZooBank LSID (Life Science Identifier) for this publication is urn:lsid:zoobank.org:pub:30F1E3C2-9D68-45A0-9CA0-E9CBA762C888.

**Figure 4:**
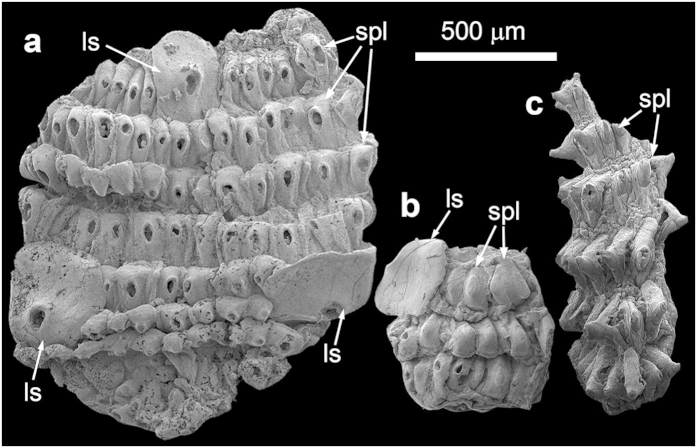
SEM images of fragmented specimens of unnamed forms. (**a,b**) Form I. (**a**) XXDW001, with 7 visible annuli and 3 large spinose sclerites. (**b**) XXDW002, with 3 visible annuli and 1 large spinose sclerite. (**c**) Form II, XXGZQ001, with 6 annuli. Scale bar applies to all images. SEM images acquired by authors.

Unnamed Forms (Fig. 4)

Additional material includes several fragmented fossils (Fig. 4) from the coeval Kuanchuanpu Formation at Xixiang section, South China (Supplementary Fig. S1)^18^,^24^,^26^,^27^, representing two unnamed forms. Form I (Fig. 4a,b) is represented by two specimens, with seven and three preserved trunk annuli, respectively. Each annulus is covered with a circlet of ~20‒40 tightly sutured small plates, which have an expanded base supporting a centrally located circular or elliptical hole likely representing a broken hollow spine. The trunk is also armored with large spinose sclerites that straddle two adjacent segments and bear a centrally located hole representing a broken hollow spine. Form II (Fig. 4c) is represented by a single specimen with six annuli, each of which is covered with a circlet of ~10–15 tightly sutured small plates with an expanded base and a centrally located hollow spine. The expanded base is defined by two lateral slopes separated by two indentations, which match the imbrication with neighboring plates. No large sclerites are present in this incompletely preserved specimen.

*Eokinorhynchus rarus* and the two unnamed forms are similar in their trunk annuli that are covered with circlets of small plates. However, they are distinguishable from each other by the presence/absence of large sclerites and the shape of small plates (e.g., spinose plates in the two unnamed forms). Thus, we interpret them as different species of a closely related group of animals.

## Discussion

**Figure 5:**
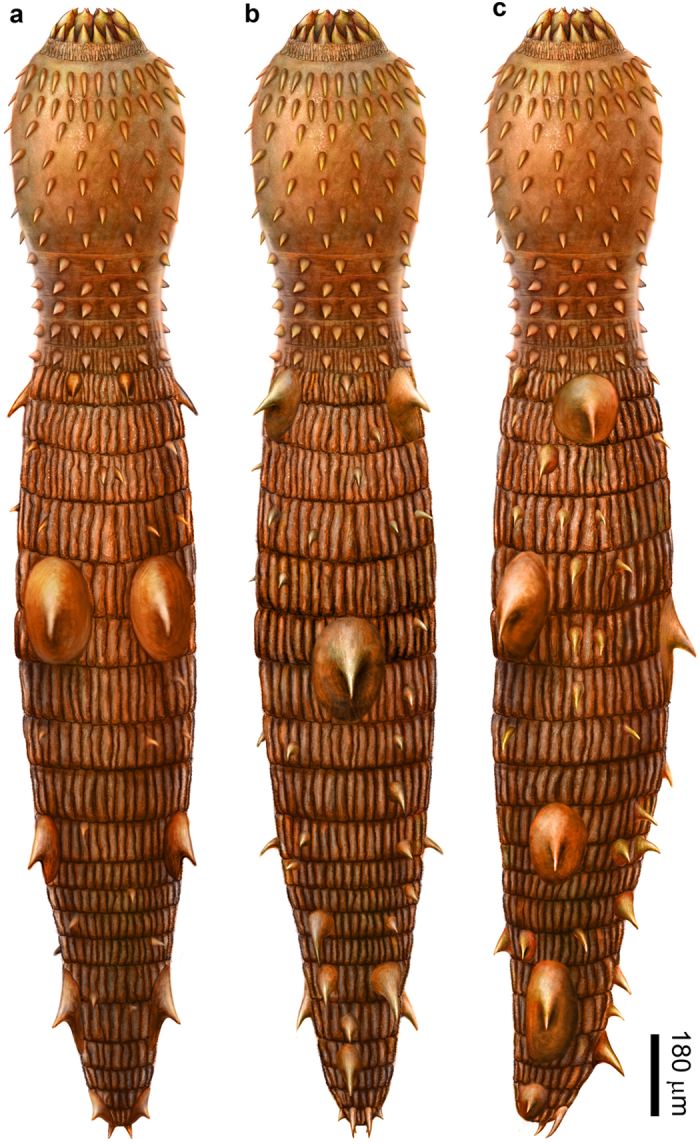
Reconstruction of *Eokinorhynchus rarus* gen. et sp. nov. (**a**–**c**) Dorsal, ventral, and right lateral views, respectively. Artwork by Mr. Dinghua Yang at Nanjing Institute of Geology and Palaeontology.

Several key morphological features of *Eokinorhynchus rarus*, as reconstructed based on available material (Fig. 5), suggest that this taxon is placed within total-group scalidophorans. An introvert with hollow scalids is considered as a scalidophoran autapomorphy, whereas nematoids have solid cuticular scalids^3^. Some panarthropods have circum-oral elements and pharyngeal teeth^28^, but their morphology (e.g., lamellate circum-oral elements vs. hollow scalids) and disposition (e.g., longitudinal rows of *Hallucigenia* aciculae do not form transverse circlets) are distinct from those of the hollow scalids of *E. rarus*. Although it is possible that circum-oral elements and pharyngeal teeth may be synapomorphies of ecdysozoans^28^, the morphology and disposition of hollow scalids and pharyngeal teeth in *E. rarus* align this taxon with scalidophorans, rather than panarthropods, nematoids, or stem-group ecdysozoans. In addition, trunk annulation is indicative of the presence of circular muscles, which are present in scalidophorans but absent in nematoids^29^. The pentaradial arrangement of introvert scalids in *E. rarus* (at least in the first two circlets of scalids) also accords with living scalidophorans but differs from the hexaradial pattern in nematoids^9^.

*Eokinorhynchus rarus* also shows intriguing similarities with extant kinorhynchs, suggesting a close phylogenetic relationship. Most importantly, the trunk epidermis of both *E. rarus* and extant kinorhynchs bears a number of hollow spines and is divided into a small number of macroannuli (11 in kinorhynchs vs. 20 or possible more in *E. rarus*), each consisting of a number of articulated plates^1^. To some extent, the multi-plate construction of *E. rarus* trunk annuli is also somewhat similar to that of palaeoscolecids which have a large number of more or less homonomous annuli and have been variously interpreted as nematomorphs^30^, stem-group priapulids^31^, or stem-group cycloneuralians^32^. But *E. rarus* and extant kinorhynchs are distinctively characterized by a small number of trunk annuli that are heteronomous in nature (i.e. due to the presence of bilaterally placed large spines, all annuli are not identical). Thus, their trunk annuli may be described as macroannuli, rather different from the large number of microannuli or wrinkles found in priapulids and many stem-group scalidophorans^2^.

However, *E. rarus* is also demonstrably different from extant kinorhynchs. It has 20 or possibly more trunk macroannuli whereas modern kinorhynchs invariably have 11. Its trunk is armored with basally expanded spines that are larger than and morphologically distinct from the filamentous trunk spines in modern kinorhynchs. In *E. rarus*, the large spines are bilaterally distributed but the small spines are randomly distributed, whereas spines in modern kinorhynchs are mostly bilaterally distributed in longitudinal rows. Although priapulid-like fossils such as *Circocosmia* and *Tabelliscolex* also have sclerites, their sclerites are plate-like structures that are bilaterally arranged in longitudinal rows along a large number of annuli^16^,^33^,^34^. On the other hand, the large sclerites of *E. rarus* and Form I are remarkably similar to disarticulated sclerites described as *Paracarinachites spinus*^35^, which may be different from the type material of *P. spinus*^36^,^37^. Similarly, the small plates in Form II resemble disarticulated sclerites described as *Kaiyangites novoli*^23^,^38^,^39^. Thus, it is possible that these small shelly fossils may represent disarticulated sclerites of kinorhynch-like animals, although it is important to bear in mind that different animals may bear similar sclerites and the same animal may have several different types of sclerites.

**Figure 6:**
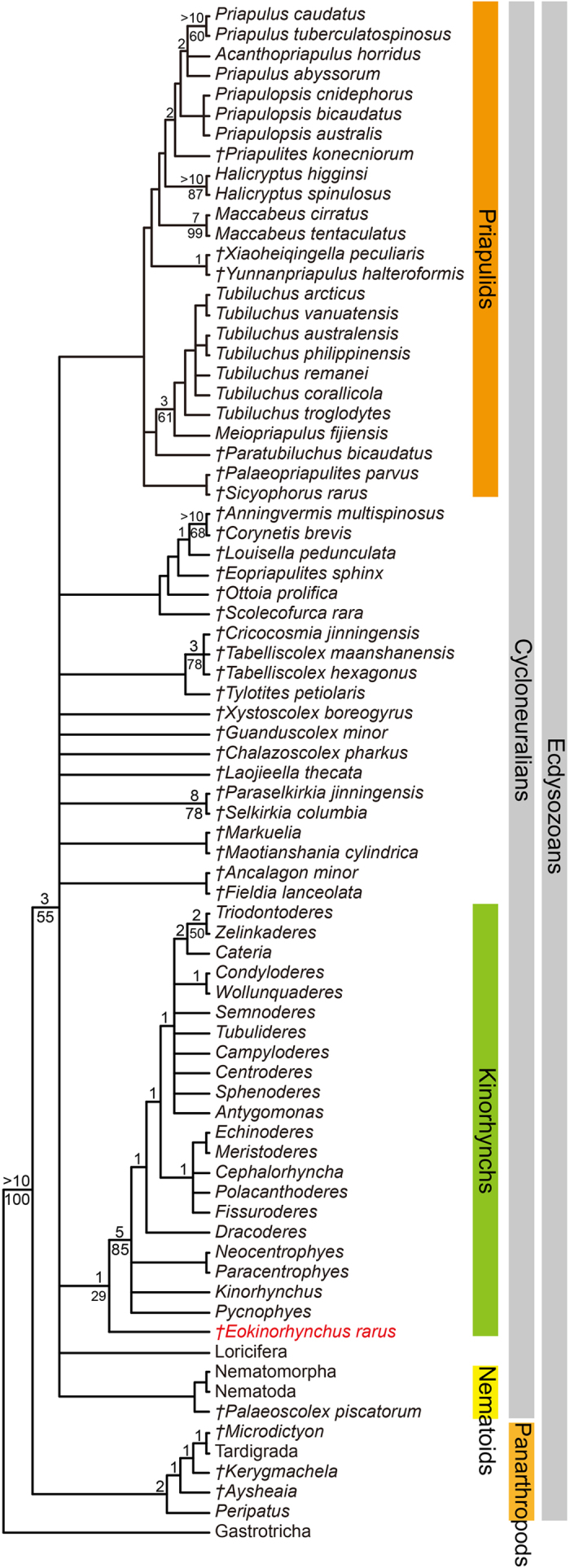
Cladogram (50% majority rule tree) showing the phylogenetic position of *Eokinorhynchus rarus* gen. et sp. nov. Daggers indicate extinct taxa. Numbers above nodes are Bremer support values (computed using TNT Bremer function with suboptimal trees up to 10 steps longer), and numbers below nodes are the Bootstrap support values (only values ≥50% are shown).

A phylogenetic analysis offers some support that *Eokinorhynchus rarus* is a stem-group kinorhynch with trunk macroannuli as a key synapomorphy (Fig. 6). Admittedly, the cladogram has relatively low resolution (with a large polytomy near the base) and most clades have relatively low Bootstrap or Bremer support. The low resolution and low support are largely due to the large amount of missing data in the data matrix (i.e., many characters describing extant taxa are not preserved in fossil taxa). Nonetheless, the paleontological data and phylogenetic interpretation presented here invite further exploration of the phosphatization taphonomic window and careful re-examination of small shelly fossils (e.g., *Paracarinachites spinus* and *Kaiyangites novoli*) in search of Cambrian kinorhynchs. If *E. rarus* and other Cambrian fossils are confirmed as stem-group kinorhynchs, then all three scalidophoran phyla must have diverged in the early Cambrian or earlier, and a Cambrian fossil record of kinorhynchs can offer fresh paleontological insights into the convergent evolution of segmentation in ecdysozoans^40^.

## Methods

### Sample preparation

Rocks were collected from the basal Cambrian Xinli Member of the Dengying Formation in northern Sichuan Province and the Kuanchuanpu Formation at the Xixiang section in southern Shaanxi Province, South China (Supplementary Fig. S1). Rock samples were first crushed into walnut-sized pieces (2 ~ 3 cm in diameter), and then dissolved in acetic acid following procedures described in Müller^41^. Rock fragments were immersed in diluted acetic acid (~10%), and residues were retrieved regularly after seven days of reaction. The residues were dried naturally, and microfossils were handpicked under a binocular microscope. Selected microfossils were mounted on aluminum stubs for scanning electron microscopy (SEM) on a LEO1530VP field-emission environmental SEM in Nanjing Institute of Geology and Paleontology and a Hitachi TM3000 desktop SEM at Virginia Tech.

### MicroCT analysis

NIGP160401 (Fig. 2) was scanned on an Xradia MicroCT scanner at Virginia Tech. The major components of the scanner include a micro-focus x-ray source, a motorized sample stage, and a motorized detector assembly. The average focal spot size is ~6 μm. The sample stage enables the rotation angle between –172.6° and 172.6°, with the minimal angular step of 0.1°. The detector assembly is equipped with five sets of x-ray optic lenses, which are named as ‘0.5×’, ‘4×’, ‘10×’, ‘20×’ and ‘40×’. These lenses provide various optical magnification levels, and different upper limits for image spatial resolution and field-of-view (FOV). Both the detector assembly and the x-ray source are mounted on the motorized linear rails. Given the type of x-ray optic lens, the source-to-sample distance and the detector-to-sample distance can be further adjusted to achieve the optimal trade-off among FOV, spatial resolution, and signal-to-noise ratio. The microCT scan parameters are summarized in Supplementary Table S2. A micro-CT scan was first conducted to acquire the tomographic images of the entire specimen. Subsequently, a ‘zoom-in’ scan was carried out to acquire the enlarged tomographic images of the head at a higher resolution. The image reconstruction was accomplished by Xradia XMReconstructor 7.0. The parameters of reconstructed images are also listed in Supplementary Table S2. Micro-CT data were processed using the software VG Studio Max 2.2 to generate three-dimensional renditions and animations (Supplementary Movies S1–4).

### Phylogenetic analysis

The data matrix was built upon Wills *et al.*^42^, Liu *et al.*^18^, and Neuhaus^1^, with the addition of new characters (characters 9, 17, 27–31, 33, 37–44, 46–53, 87, and 88) and new/revised character states (state 9 of character 8, states 2 and 3 of character 25, states 2 and 3 of character 26, states 2–5 of character 36, and state 3 of character 56) to accommodate the morphologies of *E. rarus* and modern kinorhynchs. Although the completely preserved specimens of *E. rarus* (Fig. 1, 2) could be juveniles or young adults, their morphological features are largely similar to the presumed but incompletely preserved adult specimen (Fig. 3h,i). However, caveats should be noted, because future discoveries may reveal completely preserved adult specimens that could affect the character coding of *E. rarus* adopted here. Twenty one kinorhynch genera were coded at the generic level, based on table 8 of Neuhaus^1^, representing the crown-group kinorhynchs. The codings of *Eopriapulites sphinx*^18^, Loricifera, *Aysheaia*, *Peripatus*, *Kerygmachela*, *Microdictyon*, and Tardigrada were updated. The data matrix is provided in Supplementary Table S3.

The new dataset with 77 taxa and 114 characters was analyzed using TNT with all characters weighted equally^43^. Gap mode was treated as missing, collapse rule 1 was adopted (default collapse rule in TNT), and TNT memory was set to 10,000 trees. Traditional search commands (heuristic search with 1000 random stepwise addition replicates saving 10 trees per replicate, followed by TBR branch swapping) yielded 2220 most parsimonious trees (MPTs) with tree length (TL) = 353 steps, consistency index (CI) = 0.530, and retention index (RI) = 0.845. Clade support values were calculated by means of standard bootstrap analysis implemented in TNT with 100 replications of heuristic searches with 100 interactions of random addition of taxa and holding 10 trees per interaction. Bremer support values were calculated using the TNT Bremer function with suboptimal trees up to 10 steps longer. The 50% majority rule tree is presented in Fig. 6. All data for phylogenetic analysis, including list of characters, data matrix, TNT files, and other related files are available at www.morphobank.org (Project 2209).

## Additional Information

**How to cite this article**: Zhang, H. *et al.* Armored kinorhynch-like scalidophoran animals from the early Cambrian. *Sci. Rep.* **5**, 16521; doi: 10.1038/srep16521 (2015).

## Supplementary Material

Supplementary Information

Supplementary Movie S1

Supplementary Movie S2

Supplementary Movie S3

Supplementary Movie S4
