# Supplementary material for: Armored kinorhynch-like scalidophoran animals from the early Cambrian
Source: Sci Rep. 2015 Nov 26;5:16521. doi: 10.1038/srep16521 (PMC4660871; doi:10.1038/srep16521)
Supplement: Supplementary Information [file srep16521-s1.pdf]

Armored kinorhynch-like scalidophoran animals from the early Cambrian

Huaqiao Zhang[1], Shuhai Xiao[2], Yunhuan Liu[3,4], Xunlai Yuan[4], Bin Wan[4], A. D. Muscente[2], Tiequan Shao[3], Hao Gong[5], Guohua Cao[5]

[1]Key Laboratory of Economic Stratigraphy and Palaeogeography, Chinese Academy of Sciences (Nanjing Institute of Geology and Palaeontology), Nanjing 210008, China; [2]Department of Geosciences, Virginia Tech, Blacksburg, Virginia 24061, USA; [3]College of Earth Science and Resources, Chang'an University, Xi'an 710054, China; [4]State Key Laboratory of Palaeobiology and Stratigraphy, Nanjing Institute of Geology and Palaeontology, Chinese Academy of Sciences, Nanjing 210008, China; [5]Virginia Tech – Wake Forest University School of Biomedical Engineering and Sciences, Virginia Tech, Blacksburg, VA 24061, USA

Supplementary information

1. Geological setting and age

Eokinorhynchus rarus gen. et sp. nov. was extracted from phosphatic dolostone of the lower Xinli Member of the Dengying Formation at the Xinli section in Nanjiang County, northern Sichuan Province, South China (Fig. S1). The fossil locality is the same section measured and described as Changliang section by Yang et al.[1], and as Xinli section by Steiner et al.[2,3]. The Xinli Member at Xinli section is about 26 m in thickness, and consists of, in ascending order, a basal unit of finely crystalline dolostone (2.1 m), finely crystalline phosphatic dolostone (7.9 m),

finely crystalline dolostone (12.6 m), and finely crystalline phosphatic dolostone (3.4 m)[1,2]. It is underlain by dolostone of the Dengying Formation, and overlain by black shale of the Guojiaba Formation. *E. rarus* came from the lower part of the 2nd unit, 3 m above the base of Xinli Member. Co-occurring microfossils include *Anabarites sexalox*, *Anabarites ternarius*, *Hexaconularia sichuanensis*, *Olivooides multisulcatus*, *Protohertzina unguliformis*, and *Quadrapyrgites quadratacris*.

Specimens described as Form I and Form II were extracted from phosphatic limestone of the lower Kuanchuanpu Formation at Xixiang section in southern Shaanxi Province, South China (Fig. S1). They are deposited at the University Museum of Chang'an University. The fossil locality was first described as Zhangjiagou section by Li[4], and later as Hexi section by Steiner et al.[5] and Xixiang section by Liu et al.[6]. The Kuanchuanpu Formation at Xixiang section is about 22 m in thickness, and consists of, in ascending order, a basal unit of light gray microsparitic limestone (0.8 m), phosphatic limestone (3.2 m), thick-bedded dark microsparitic limestone (17.4 m), and thin-bedded dolomitic limestone (0.6 m)[4]. It is underlain by dolostone of Dengying Formation, and overlain by black shale of the Guojiaba Formation. Fossils of Forms I and II came from the lower part of the 2nd unit, 1.5 m above the base of Kuanchuanpu Formation. Co-occurring microfossils include *Protohertzina anabarica*, *Olivooides multisulcatus*, *Olivooides mirabilis*, *Quadrapyrgites quadratacris*, and a stem scalidophoran *Eopriapulites sphinx*[6].

Co-occurring small shelly fossils indicate that the two horizons yielding the present material are part of the *Anabarites trisulcatus*-*Protohertzina anabarica* Assemblage Zone[3], which

belongs to the Fortunian Stage[7]. The age of Fortunian Stage is considered to be between 541.0 and 529.0 Ma[7], and the *Anabarites trisulcatus–Protohertzina anabarica* Assemblage Zone is estimated to be approximately 535 Ma[3,5].

2. Character List

Description of morphological characters, revised from Wills et al.[8], Liu et al.[6], and table 8 of Neuhaus[9].

Anterior

1. Degree to which the introvert can be invaginated: no introvert, or introvert not invaginable at all (0), partially invaginable (i.e., part of zone I) (1), completely invaginable into the trunk (i.e., to the base of zone I) (2).

2. Anterior branched frontal appendages: absent (0), present (1).

3. Scalids (nonspecific and sensu lato, including both scalids and presumed scalid derivatives): absent (0), present and composed exclusively of cuticle (1), present and with cuticle limited to a thin outer covering (2). Schmidt-Rhaesa[10] has demonstrated that, in comparison to the scalids of Scalidophora (Kinorhyncha +Loricifera+Priapulida), the scalids of nematoids (Nematoda+Nematomorpha) are composed exclusively of cuticle. The collapse and folding of the scalids in *Markuelia* indicates that they are hollow structures and, thus, not wholly composed of cuticle.

4. Mouth cone: absent (0), present but non-inversible (1), present and inversible (2). The mouth cone is the eversible (though not necessarily inversible) upstanding anterior limit of the

pharynx.

5. Zone I: unarmed (0), armed (1).

6. Arrangement of zone I armature: not in rows (0), discrete parallel longitudinal rows (1), rows aligned diagonal to the anterior-posterior axis of the animal (2).

7. Radial arrangement of armature: pentaradial (0), hexaradial (1).

8. Morphology of zone I armature: papillae (0), simple spines (1), hooks or spinose hooks (2), conical scalids (3), telescopiform scalids (4), curved scalids and dentoscalids (5), complex scalids (6), glandular scalids, trifid spines, sensory spines, and double, tentaculite scalids (7), scalids with pectinate hood (8), spinoscalids, trichoscalids, and clavoscalids (kinorhynchs and loriciferans) (9).

9. Number of trichoscalids of Zone I: 0 (0), 6 (1), 9 (2), 14 (3).

10. Number of elements comprising the first three circlets and, hence, defining the number of longitudinal rows of elements on the introvert: none (0), < 20 (1), 25 (2), > 25 (3). In all extant priapulids there are eight elements in the most proximal circlet of the introvert, their number and position corresponding to innervation derived from the circumpharyngeal brain.

11. Sequence of zone I elements: elements as a single series (all elements identical or with differing morphologies) (0), elements organized into two or more transverse bands or series, possibly with different element morphologies within each series, but the sequence of morphologies being comparable between subsequent series (1).

12. Basal circlet of zone I armature separated from more anterior by a constriction (as in loriciferans) or by insertion of longitudinal or circular muscles (kinorhynchs): absent (0),

present (1).

13. Number of elements in the proximal circlet of zone II: none or zone unarmed (0), eight (1), more than eight (2).

14. Elongate spines at the base of Zone II: absent (0), present (1).

15. Number of circlets of zone III armature: none or unarmed (0), one to four (1), six to fourteen (2), sixteen or more (3).

16. Morphology of proximal circlets of zone III armature ("teeth"): spines or papillae (0), multispinose (1), multispinose but massively reduced (2), hooks (3), conical with a fringe of spines (4), sclerotized trabeculae (5), pectinate (6), conical papillae terminating in a long spine (prickle) (7), oral styles (8).

17. Number of outer oral styles: 1 (0), 2 (1), 3 (2), 4 (3), 6 (4), fused (5)

18. Morphology of middle circlets of zone III armature (teeth): absent (0), spines (of any length) or papillae (1), multispinose (reduced or otherwise) (2), pectinate (3).

19. Morphology of the distal circlets of zone III armature (teeth): absent (0), spines (of any length) (1), multispinose (reduced or otherwise) (2), pectinate (3).

20. Number of elements in first circlet of pharyngeal armature (base of zone III): elements absent (0), first circlet of 5 elements (1), first circlet of 8-10 elements (2), first circlet of numerous elements (more than 10) (3).

21. Number of proximal, pentagonal circlets in zone III of the proboscis: none (0), five (1), six (2), seven (3).

22. Width of zone III relative to zone II: zone III less than twice the width of zone II (0), zone III

equal to or greater than twice the width of zone II (1).

23. Width of the distal portion of zone III: distal zone III parallel to proximal zone III or tapering gradually (0), distal zone III expanded into a bulb (1).

24. Eversibility of zone III: zone III completely eversible (0), zone III incompletely eversible, but eversible beyond the proximal teeth (1), zone III normally eversible only as far as the proximal teeth (2).

25. All zone III elements of approximately equal size (0), zone III elements decreasing regularly in size from the posterior to the anterior (anteriormost elements less than half the size of the posteriormost) (1), zone III elements increasing in size from posterior to anterior (2), zone III elements first increasing and then decreasing in size from posterior to anterior (3).

Neck

26. Armament of neck or neck region: none (0), cuticular plates (as in larvae/juveniles) (1), scalids (2), cuticular plates + scalids (3).

27. Placids: absent (0), present (1).

28. Placids fused with macroannulus 1: no (0), yes (1).

29. Placids tripartite anteriorly: no (0), yes (1).

30. Placids with knobby projections: no (0), yes (1).

31. Number of placids: 6 (0), 7 (1), 8 (2), 9 (3), 14 (4), 16 (5).

Trunk

32. Aspect ratio of body length to width in adult: <10 (0), 10-20 (1), >20 (2).

33. Trunk cuticles with longitudinal striation pattern: absent (0), present (1).

34. Paired, lateral, locomotory appendages: absent (0), present (1).

35. Annulation: absent (0), homonomous (1), heteronomous (2). Budd[11] has drawn comparison between the patterns of cuticular ornament in association with the trunk annulae in lobopods and palacoscolecids. However, although palaeoscolecids exhibit an alternating pattern of cuticular ornament[12] and although this varies from one body region to another, adjacent annulae are identical; they exhibit homomous annulation. This contrasts with the condition in lobopods where adjacent annulae vary both in terms of their axial length and their cuticular ornamentation.

36. Number of trunk annuli: <7 (0), 7-11 (1), 12-29 (2), 30-60 (3), 61-120 (4), 140 or more (5).

37. Macroannuli: absent (0), present (1). Macroannuli refer to divisions (annular zones or somites) of the trunk. Macroannuli are wider and thus fewer in numbers than microannuli, and they are present in some arthropods and kinorhynchs[13].

38. Number of trunk macroannuli: <=11 (0), >11 (1).

39. Trunk macroannulus 1: ring-like (0), ring-like with slight mid-ventral and mid-dorsal anterior indentation (1), 1 sternal + 1 tergal plate (2), 3 sternal + 1 tergal plate (3), clamshell-like, anterior, broad incision with large triangular mid-ventral placid (4), clamshell-like, anterior, narrow incision with narrow, elongate mid-ventral placid (5).

40. Trunk macroannulus 2: ring-like (0), 1 tergal plate with mid-ventral junction (1), 1(mid)sternal + 1 tergal plate (2), 2 sternal + 1 tergal plates (3).

41. Trunk macroannuli 3 to 4: ring-like (0), 2 sternal + 1 tergal plates (1), 1 midsternal + 1 tergal plate (2), 1 tergal plate with mid-ventral junction (3).

42. Trunk macroannulus 5: ring-like (0), 2 sternal + 1 tergal plates (1), 1 mid-sternal + 1 tergal plate (2), 1 tergal plate with mid-ventral junction (3).

43. Trunk macroannulus 6: ring-like (0), 2 sternal + 1 tergal plates (1), 1 mid-sternal + 1 tergal plate (2), 1 tergal plate with mid-ventral junction (3).

44. Trunk macroannuli 8 to 10: ring-like (0), 2 sternal + 1 tergal plates (1), 1 tergal plate with mid-ventral junction (2).

45. Trunk spines, fine spines, or setae in adult: absent (0), present (1).

46. Cuspidate spines on any macroannulus: absent (0), present (1).

47. Latero-ventral spine on trunk macroannulus 1: absent (0), present (1).

48. Acicular lateral spines at least on some trunk macroannuli 2–9: absent (0), present (1).

49. Short spine ventro-medially, ventro-laterally, sub-dorsally, and latero-dorsally in several trunk macroannuli 4–10: absent (0), present (1).

50. Lateral terminal spine on terminal macroannulus: absent (0), present (1).

51. Lateral terminal accessory spine on terminal macroannulus: absent (0), present (1).

52. Mid-terminal spine on terminal macroannulus: absent (0), present (1).

53. Short mid-terminal spinose process on terminal macroannulus: absent (0), present (1).

54. Trunk papillae in adult: absent (0), present and simple (1), extremely long and arranged into two discrete, longitudinal rows (2).

55. Trunk sclerites in adult: absent (0), present and small to medium in size (1), present and

greatly enlarged (2).

56. Trunk sclerites: absent (0), in two longitudinal rows (1), in annular rings (2), bilaterally arranged, but not in rows (3).

57. Trunk tumuli: absent (0), present (1).

58. Trunk tubuli: absent (0), present (1).

59. Trunk tubercles of soft, coriaceous consistency: absent (0), present (1).

60. Flosculi, N-flosculi or sensory spots: absent (0), flosculi present with between 7 and 11 petals (1), flosculi present and invariably with 8 petals (2).

61. Clavulae: absent (0), present (1).

62. Tube: Absent (0), tube composed of plant debris: absent (1), tube flattened, annulated, tapering and chitinous (2).

Posterior region

63. Arc or ring of posterior spines or hooks (distinct from the bilaterally symmetrical structures present in *Markuelia* and many palaeoscolecids): absent (0), present (1).

64. Posterior ring papillae: absent (0), present (1).

65. Eversible bursa: absent (0), present (1).

66. Posterior abdomen greatly extensible: no (0), yes (1).

67. Position of the anus: anus terminal, whether within bursa or otherwise (0), anus in posterolateral or posteroventral surface of the abdomen (1).

68. Posterior tubulae or setae: absent (0), present (1).

69. Caudal appendage(s): absent (0), present but less than the length of the body (1), present and up to three times the lenth of the body (2). Harvey *et al.*[14] coded a caudal appendage as present in *Kerygmachela*. However, other characters contingent on the presence of a caudal appendage are coded as inapplicable for *Kerygmachela* by these authors (as for taxa lacking a caudal appendage). We therefore code an appendage as absent in *Kerygmachela*.

70. Division of caudal appendage(s) or tail: undivided (0), pseudosegmented (1).

71. Caudal appendage(s): single and positioned terminally (0), single and positioned dorso-medially (1), bicaudal as in some species of *Priapulopsis* and *Paratubiluchus* (2).

72. Surface of caudal appendage: smooth (0), vesiculae (1), large warts (2).

73. Terminally posterior spines, hooks or cones of basal diameter >20% trunk diameter: absent (0), present and arranged in pairs about the sagittal plane (1). This definition of "spines, hooks or cones" excludes the posterior body bifurcations of *Aysheaia*, *Kerygmachela*, tardigrades, some nematomorphs, some kinorhynchs and gastrotrichs. The paired, peri-anal structures in *Halicryptus* and *Maccabeus* are much smaller relative to body width.

74. Posterior warts: absent (0), small (as in *Halicryptus*) (1), large (as in *Priapulus*) (2).

Gut

75. Polythyridium: absent (0), present (1).

Nervous system

76. Ventral nerve cord unpaired throughout its length: absent (0), present (1). Living priapulids

possess unpaired ventral nerve cords, whereas gastrotrichs, onychophorans and loriciferans possess ventral nerve cords that are paired throughout their length, and the ventral nerve cords of nematomorphs and nematodes divide at points along their length[10]; the situation in kinorhynchs is unresolved (paired according to Kristensen and Higgins[15]; unpaired according to Neuhaus[16]. The condition in *Ottoia* is common to extant priapulids[12].

77. Ventral nerve cords merge caudally: absent (0), present (1).

78. Dorsal nerve cord unpaired: absent (0), present (1).

79. Circumpharyngeal brain: absent (0), present (1).

80. Brain with anterior posterior sequence of perikarya-neuropil-perikarya: absent (0), present (1). Lemburg[17] recognized this as a synapomorphy of Introverta (Nematoda + Nematomorpha + Kinorhyncha + Loricifera + Priapulida).

81. Apical part of the brain composed of perikarya: absent (0), present (1) (Lemburg[17]).

82. Pharyngeal nervous system composed of numerous tooth ganglia connected by a diagonal nerve net: absent (0), present (1). Lemburg[17] recognizes the presence of this character as a synapomorphy of (extant) Priapulida.

83. Two rings of introvert retractors attached through the collar-shaped brain: absent (0), present (1), proposed by Nielsen[18] as a synapomorphy of kinorhynchs, loriciferans and extant priapulids.

Larval and Developmental characters

84. Developmental mode: direct (0), biphasic (1).

85. Loricate stage: absent (0), present (1).

86. Cuticle of the larva/juvenile dorso-ventrally flattened (at least in older stages), with six lateral plates in-folded and accordion like. Cuticle of the lorica thickened in dorsal and ventral plates (at least) with sculpture of four to six longitudinal rows of narrow, rectangular fields: absent (0), present (1).

87. Cuticles of the annuli divided into plates, especially in late juveniles or adults: absent (0), present (1).

88. Number of plates in each annulus: 1~4 (0), 10~40 (1).

89. Larvae/juveniles with six long pharynx retractor muscles: absent (0), present (1). Lemburg[17] recognized the presence of this character as a synapomorphy of (extant) Priapulida. However, it has since been demonstrated that the larvae of nematomorphs also possess six pharyngeal retractor muscles[19,20].

90. Teeth of second circle of the larvae/juveniles with very small median denticle: absent (0), present (1). Lemburg[17] recognizes the presence of this character as a synapomorphy of Megaintroverta (*Priapulopsis* + *Acanthopriapulus* + *Priapulus*).

91. Division of the body into a distinct proboscis and abdomen in larva/juvenile: absent (0), present (1).

Reproduction

92. Cloaca in both sexes: absent (0), present (1).

93. Protonephridia: absent (0), present but do not flow into the gonoduct and are not integrated

into the gonad (1), present and flow into the gonoduct and/or are integrated into the gonad (2). Protonephridia are considered an apomorphy of the Bilateria[21] and are present in gastrotrichs, kinorhynchs, loriciferans and extant priapulids, but absent, presumably secondarily, from onychophorans, nematodes and nematomorphs. Lemburg[17] recognized state 2 as a synapomorphy of lorciferans and extant priapulids.

94. Urogenital system attached to the body wall by a ligament: absent (0), present (1). Lemburg[17] recognized this as a synapomorphy of lorciferans and extant priapulids.

95. Spermatozoa with a flagellum: absent (0), present (1). The presence of a flagellum in spermatozoa is a metazoan symplesiomorphy, but a flagellum is lacking from the spermatozoa of nematodes and nematomorphs[10].

96. Genital field extremely simplified: no (0), yes (1).

97. Copulatory organs: absent (0), present (1).

98. Perigenital setae: absent or reduced (0), prominent (1).

Cuticle

99. Cuticle predominantly containing collagen: absent (0), present (1).

100. Distribution of chitin in the cuticle: none (0), predominantly within the middle cuticle layer (exocuticle) (1), predominantly within the lowermost cuticle layer (endocuticle) (2).

101. Cuticle with homogeneous layer at surface/beneath epicuticle: absent (0), present (1).

102. Cuticle with middle layer of distinct composition: absent (0), present (1).

103. Cuticle with radially striated/vertical canal middle layer: absent (0), present (1).

104. Cross-wise fibres in cuticle: absent (0), present (1). (Reported in *Meiopriapulus* by Storch et al.[22].

105. Large helical fibres in the cuticle: absent (0), present and composed of platy subunits (1), present and composed of unpaired fibrils (2), present and composed of paired, wound fibrils (3).

106. Cuticle surface with ornament of tessellating polygons: absent (0), present (1).

General body plan

107. Division of the body into a distinct proboscis and abdomen in adult: absent (0), present (1).

108. Terminal mouth: absent (0), present (1).

109. Percentage of animal length taken up by the introvert: None (no introvert) (0), 2% (as in *Halicryptus higginsi*) (1), between 2% and 30% (2), 30 to 50% (3). Lemburg[17] recognizes a very large introvert (state 3) as a synapomorphy of Megaintroverta (*Priapulopsis* + *Acanthopriapulus* + *Priapulus*).

110. Voluminous primary body cavity: absent (0), present (1). Lemburg[17] recognizes the presence of this character as a synapomorphy of (extant) Priapulida.

111. Movement by peristaltic movement of the pharynx/introvert: absent (0), present (1).

112. Pharyngeal lumina: round (0), triradiate (1).

113. Circular body musculature: absent (0), present (1). Circular body musculature is present in all taxa of nemathelminth grade except nematodes and nematomorphs, and its absence has been considered both secondary and a synapomorphy of Nematoida[10].

114. Nucleation of "peritoneal" membrane: membrane without nuclei or simply with amoebocytes in association with the surface (0), membrane containing scattered nuclei (1).

Supplementary references:

1. Yang, X., He, Y. & Deng, S. On the Sinian–Cambrian boundary and the small shelly fossil assemblages in Nanjiang Area, Sichuan. *Bull. Chengdu Inst. Geol. Min. Res., Chin. Acad. Geol. Sci.* **4**, 91-110 (1983).
2. Steiner, M., Li, G., Qian, Y. & Zhu, M. Lower Cambrian Small Shelly Fossils of northern Sichuan and southern Shaanxi (China), and their biostratigraphic importance. *Geobios* **37**, 259-275 (2004).
3. Steiner, M., Li, G., Qian, Y., Zhu, M. & Erdtmann, B.-D. Neoproterozoic to early Cambrian small shelly fossil assemblages and a revised biostratigraphic correlation of the Yangtze Platform (China). *Palaeogeogr. Palaeoclimatol. Palaeoecol.* **254**, 67-99 (2007).
4. Li, Z. The discovery and its significance of small shelly fossils in Hexi area, Xixiang, Shaanxi. *Geol. Shaanxi* **2**, 73-77 (1984).
5. Steiner, M., Qian, Y., Li, G., Hagadorn, J. W. & Zhu, M. The developmental cycles of early Cambrian Olivooidae fam. nov. (?Cycloneuralia) from the Yangtze Platform (China). *Palaeogeogr. Palaeoclimatol. Palaeoecol.* **398**, 97-124 (2014).
6. Liu, Y., Xiao, S., Shao, T., Broce, J. & Zhang, H. The oldest known priapulid-like scalidophoran animal and its implications for the early evolution of cycloneuralians and ecdysozoans. *Evol. Dev.* **16**, 155-165 (2014).
7. Peng, S., Babcock, L. E. & Cooper, R. A. The Cambrian Period. In *Geological Time Scale 2012* (eds Gradstein, F. M., Ogg, J. G., Schmitz, M. & Ogg, G.), 437-488 (Elsevier, Oxford, 2012).
8. Wills, M. A., Gerber, S., Ruta, M. & Hughes, M. The disparity of priapulid, archaeopriapulid and palaeoscolecid worms in the light of new data. *J. Evol. Biol.* **25**, 2056-2076 (2012).
9. Neuhaus, B. Kinorhyncha (=Echinodera). In *Handbook of Zoology: Gastrotricha, Cycloneuralia and Gnathifera, Volume 1: Nematomorpha, Priapulida, Kinorhyncha, Loricifera* (ed Schmidt-Rhaesa, A.), 181-348 (Walter de Gruyter GmbH, Berlin/Boston, 2013).
10. Schmidt-Rhaesa, A. Phylogenetic relationships of the nematomorpha - a discussion of current hypotheses. *Zool. Anz.* **236**, 203-216 (1998).
11. Budd, G. E. Arthropods as ecdysozoans: the fossil evidence. In *The New Panorama of Animal Evolution: Proceedings of the XVIII International Congress of Zoology, Athens, Greece, September 2000* (eds Legakis, A., Sfenthourakis, S., Polmeni, R. & Thessalou-Leggaki, M.), 479-487 (Pensoft, Sofia, 2003).
12. Conway Morris, S. Fossil priapulid worms. *Spec. Pap. Palaeontol.* **20**, 1-95 (1977).
13. Budd, G. E. Why are arthropods segmented? *Evol. Dev.* **3**, 332-342 (2001).
14. Harvey, T. H. P., Dong, X. P. & Donoghue, P. C. J. Are palaeoscolecids ancestral ecdysozoans? *Evol. Dev.* **12**, 177-200 (2010).
15. Kristensen, R. M. & Higgins, R. P. Kinorhyncha. In *Microscopic Anatomy of Invertebrates, Volume 4: Aschelminthes* (eds Harrison, F. W. & Rupert, E. E.), 377-404 (Wiley-Liss, New York, 1991).
16. Neuhaus, B. Ultrastructure of alimentary canal and body cavity, ground pattern and phylogentic

relationships of the Kinorhyncha. *Microfauna Mar.* **9**, 61-156 (1994).

17. Lemburg, C. Ultrastrukturelle Untersuchungen an den Larven von *Halicryptus spinulosus* und *Priapulus caudatus*. Hypothesen zur Phylogenie der Priapulida und deren Bedeutung für die Evolution der Nemathelminthes. Universität Göttingen (1999).
18. Nielsen, C. *Animal Evolution: Interrelationships of the Living Phyla* (Oxford University Press, Oxford, 2012).
19. Kristensen, R. M. Do the ultrastructural investigations of Loricifera and Tardigrada support the clade Ecdysozoa? In *The New Panorama of Animal Evolution: Proceedings of the 18th International Congress of Zoology* (eds Legakis, A., Sfenthourakis, S., Polymeni, R. & Thessalou-Legaki, M.), 467-477 (Pensoft Publishers, Sofia, 2003).
20. Müller, M. C. M., Jochmann, R. & Schmidt-Rhaesa, A. The musculature of horsehair worm larvae (*Gordius aquaticus*, *Paragordius varius*, Nematomorpha): F-actin staining and reconstruction by cLSM and TEM. *Zoomorphology* **123**, 45-54 (2004).
21. Ax, P. *Multicellular Animals: A New Approach to the Phlylogenetic Order in Nature. Volume 1* (Springer-Verlag, Berlin, 1996).
22. Storch, V., Higgins, R. P. & Morse, M. P. Ultrastructure of the body wall of *Meiopriapulus fijiensis* (Priapulida). *Trans. Amer. Micros. Soc.* **108**, 319-331 (1989).

Supplementary Figure, Table, and Dataset Captions:

Supplementary Figure S1 | Location map and stratocolumns of the Xinli section in northern Sichuan Province and the Xixiang section in southern Shaanxi Province, South China. Fossil horizons are denoted by stars. Scale bar applies to both stratocolumns. Maps and stratocolumns created using Adobe Illustrator.

Supplementary Figure S2 | SEM images of *Eokinorhynchus rarus* gen. et sp. nov., holotype, NIGP160400. (a–d) Dorsal, right lateral, ventral, and left lateral views, respectively. Numbers mark introvert and neck scalids. Scale bar applies to all images. SEM images taken by authors.

Supplementary Figure S3 | SEM (a–e) and microCT images (f–i) of *Eokinorhynchus rarus* et sp. nov., paratype, NIGP160401. (**a**) Anterior region. (**b**) Posterior end. (**c**) Posterior left part. (**d**) Posterior right part. (**e**) Posterior ventral part. (**f–i**) microCT images, not to scale. (**f**) Virtual coronal section of the head. (**g**) Virtual saggital section of the head. (**h**) Virtual transverse section of the head. (**i**) Virtual transverse section at 1ls (the 1st pair of large sclerites). Scale bar between (**d–e**) applies to (**c–e**). SEM and microCT images taken by authors.

Supplementary Movie S1 | MicroCT volume rendition of the paratype NIGP160401 of *Eokinorhynchus rarus* gen. et sp. nov. The five pairs of large spinose sclerites are highlighted to show their articulation pattern. Movie generated by authors.

Supplementary Movie S2 | MicroCT movie of the paratype NIGP160401 of *Eokinorhynchus rarus* gen. et sp. nov. Slice images parallel to the saggital plane, starting from the left side. Movie generated by authors.

Supplementary Movie S3 | MicroCT movie of the paratype NIGP160401 of *Eokinorhynchus rarus* gen. et sp. nov. Slice images parallel to the coronal plane, starting from the dorsal side. Movie generated by authors.

Supplementary Movie S4 | MicroCT movie of the paratype NIGP160401 of *Eokinorhynchus rarus* gen. et sp. nov. Slice images parallel to the transverse plane, starting from the anterior end. Movie generated by authors.

Supplementary Table S1 | Measurements of selected specimens.

Supplementary Table S2 | Parameters for micro-CT scan and image reconstruction.

Supplementary Table S3 | Data matrix for cladistics analysis. This data matrix is built upon Wills et al.[8], Liu et al.[6], and Neuhaus[9].

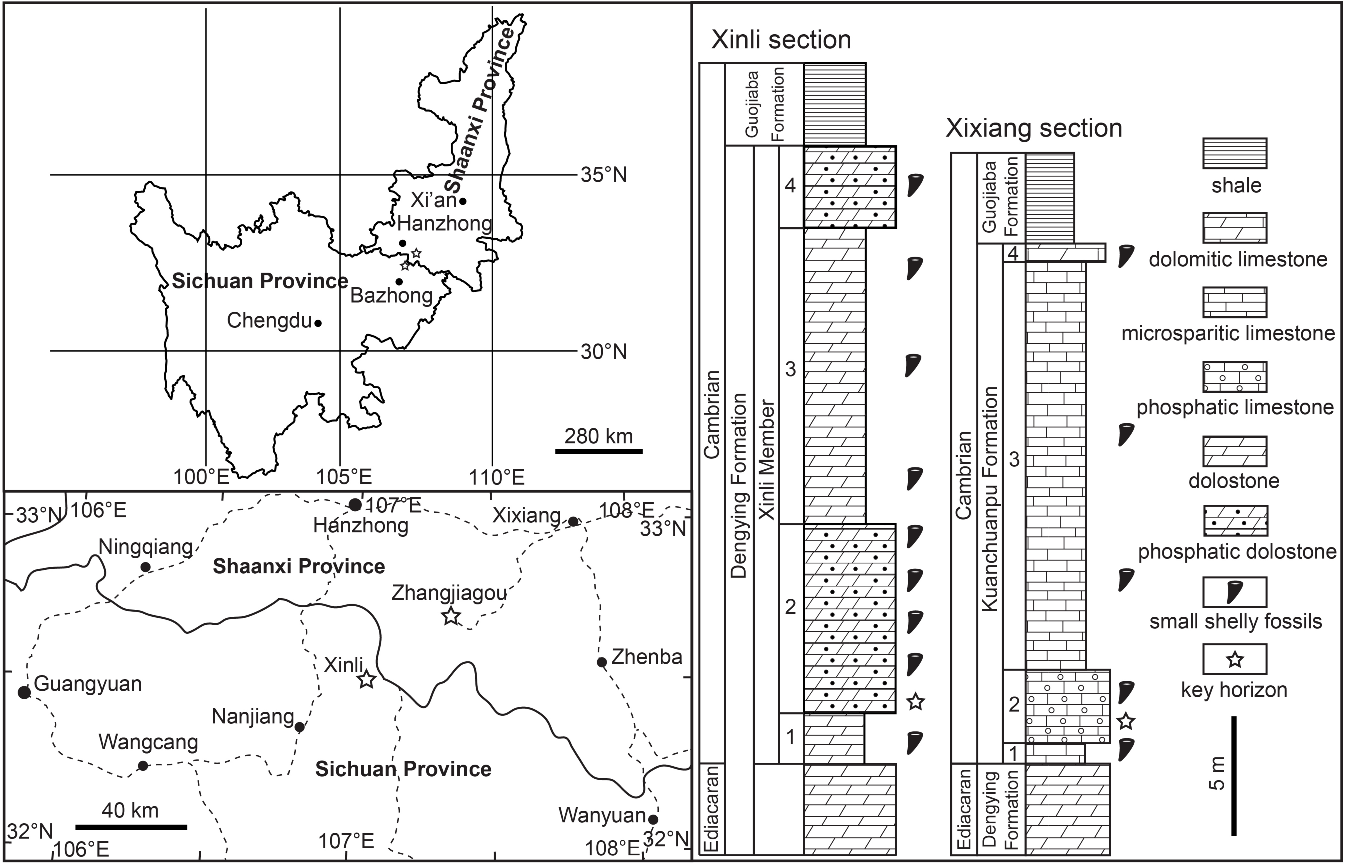

Shaanxi Province
35°N
Xi'an
Hanzhong
Sichuan Province
Bazhong
Chengdu
30°N
280 km
100°E
105°E
110°E
33°N
106°E
107°E
Hanzhong
Xixiang
108°E
33°N
Ningqiang
Shaanxi Province
Zhangjiagou
Zhenba
Xinli
Guangyuan
Nanjiang
Wangcang
Sichuan Province
40 km
Wanyuan
32°N
106°E
107°E
108°E
32°N
Xinli section
Cambrian
Ediacaran
Guojiaba Formation
Dengying Formation
Xinli Member
4
3
2
1
Xixiang section
Guojiaba Formation
Cambrian
Kuanchuanpu Formation
Ediacaran
Dengying Formation
shale
dolomitic limestone
microsparitic limestone
phosphatic limestone
dolostone
phosphatic dolostone
small shelly fossils
key horizon
5 m

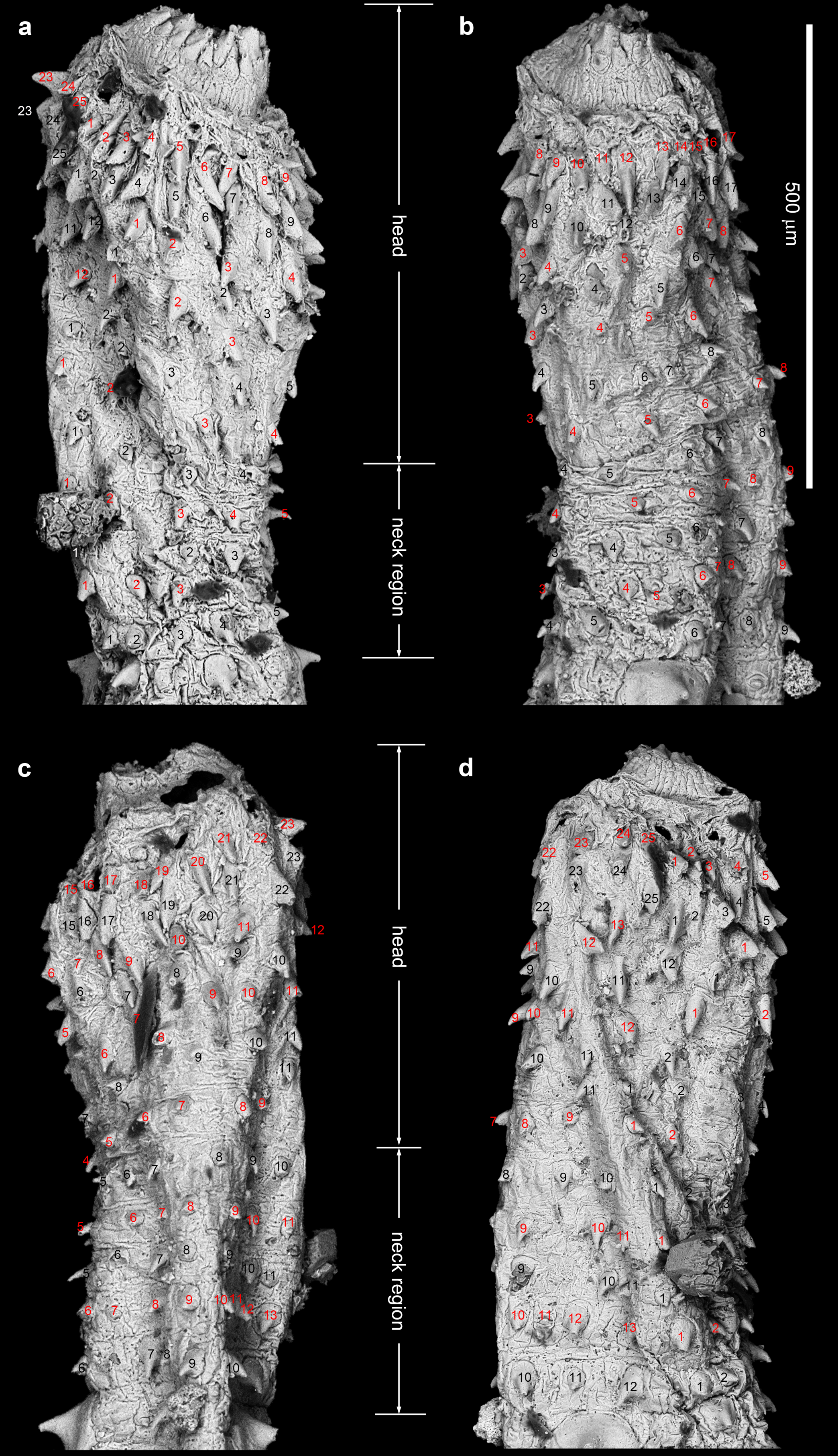
a
b
500 µm
head
neck region
c
d
head
neck region

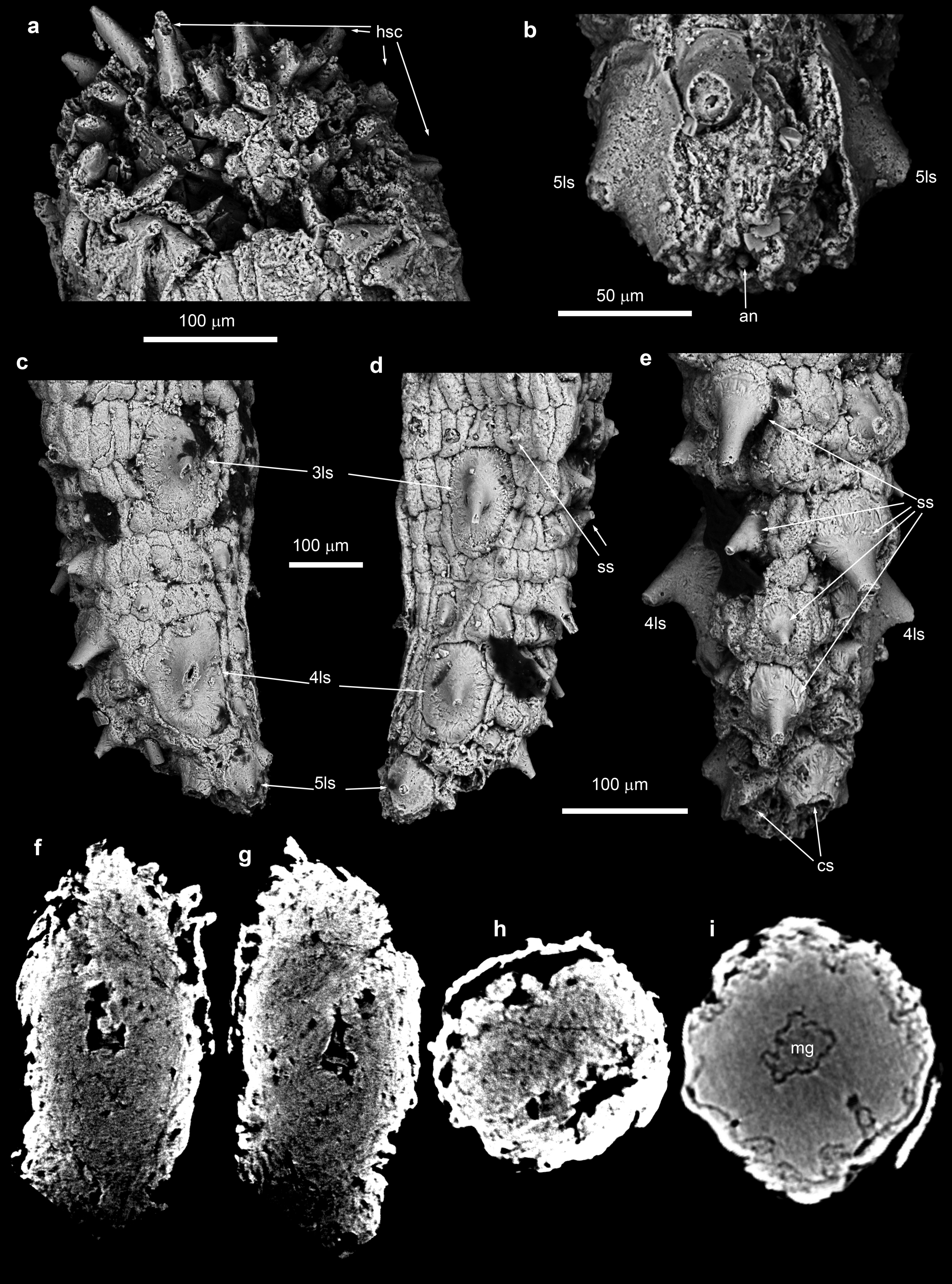

a
hsc
100 μm
b
5ls
5ls
50 μm
an
c
3ls
4ls
5ls
d
100 μm
ss
e
ss
4ls
4ls
100 μm
cs
f
g
h
i
mg

Supplementary Table S1. Measurements of selected specimens.

**Holotype of *Eokinorhynchus rarus* gen. et sp. nov. (Fig. 1)**					
Pharyngeal teeth: outermost circlet	Conical	16 in number	34 μm long	~21 μm wide at base	
Pharyngeal teeth: 2nd circlet		8 in number	46 μm long	~35 μm wide at base	
Zone 2	Cylindrical		40 μm long	170 μm in diameter	
Introvert	Cylindrical		370 μm long	287 μm wide	
Introvert scalids: 1st (anteriormost) circlet	Conical	25 in number	80 μm long	20 μm wide at base	
Introvert scalids: 2nd circlet		25 in number	72 μm long	21 μm wide at base	
Introvert scalids: 3rd circlet		13 in number	60 μm long	21 μm wide at base	
Introvert scalids: 4th circlet		12 in number	57 μm long	22 μm wide at base	
Introvert scalids: 5th circlet		12 in number	52 μm long	24 μm wide at base	
Introvert scalids: 6th circlet		11 in number	36 μm long	18 μm wide at base	
Introvert scalids: 7th circlet		9 in number	31 μm long	16 μm wide at base	
Neck scalids: 1st circlet	Conical with slightly expanded base	10 in number	28 μm long	19 μm wide at base	
Neck scalids: 2nd circlet		11 in number	36 μm long	20 μm wide at base	
Neck scalids: 3rd circlet		11 in number	35 μm long	23 μm wide at base	
Neck scalids: 4th circlet		13 in number	33 μm long	27 μm wide at base	
Neck scalids: 5th circlet		12 in number	28 μm long	29 μm wide at base	
**Other data of type specimens of *Eokinorhynchus rarus* gen. et sp. nov. (Figs. 1, 2, 3a–g)**					
Holotype (Fig. 1) length		1.41 mm (with pharynx totally everted)			
Larger paratype (Fig. 2) length		1.9 mm (with introvert partially inverted)			
Trunk: small plates	Rectangular	20–40 in each circlet	~32–100 μm long	~23 μm wide	
Trunk: small spines	Conical, some with slightly expanded base	Variable in number	~5–103 μm long	~5–109 μm wide at base	

Trunk: 1st pair of large spinose sclerites	Conical spine with strongly expanded base: similar to disarticulated small shelly fossils (incorrectly) described as *Paracarinachites spinus*	Located on s1–2	Spine: 71–81µm long	Spine: 42–68µm wide at base	Expanded base: 106–178µm wide
Trunk: 2nd pair of large spinose sclerites		Located on s5–6	Spine: 47–70 µm long	Spine: 50–53 µm wide at base	Expanded base: 125–157 µm wide
Trunk: single midventrally located large spinose sclerite		Located on s6–7	Spine: 68–76 µm long	Spine: 76–84 µm wide at base	Expanded base: 177–192 µm wide
Trunk: 3rd pair of large spinose sclerites		Located on s10–11	Spine: 44–75 µm long	Spine: 39–62 µm wide at base	Expanded base: 87–137 µm wide
Trunk: 4th pair of large spinose sclerites		Located on s15–17	Spine: 39–71 µm long	Spine: 48–68 µm wide at base	Expanded base: 86–169 µm wide
Trunk: 5th pair of large spinose sclerites		Located on s19–20	Spine: 20–33 µm long	Spine: 19–43 µm wide at base	Expanded base: 38–90 µm wide
Trunk: caudal spines	Conical, with slightly expanded base	2 pairs; located on s20	38–42 µm long	38–54 µm wide at base	
**Data of the additional material of *Eokinorhynchus rarus* gen. et sp. nov. (Fig. 3h, i)**					
The largest spinose sclerites on the distorted trunk part			Spine: 144 µm long	Spine: 176 µm wide at base	Expanded base: 294 µm wide
**Form I (Fig. 4a, b)**					
Trunk: small plates	Rectangular base supporting a broken spine	20–40 in each circlet	~180 µm long	~110 µm wide	
Trunk: large spinose sclerites					Expanded base: ~390 µm wide
**Form II (Fig. 4c)**					
Trunk: small plates	Expanded and indented base supporting a hollow spine: similar to disarticulated small shelly fossils described as *Kaiyangites novolis*	10–15 in each circlet	~240 µm long	95 µm wide	

Supplementary Table S2. Parameters for micro-CT scan and image reconstruction.

Scans	Entire specimen	Head only
X-ray tube voltage (kV)	40	35
X-ray tube power (W)	8	7
Exposure time per projection image (s)	55	35
Source-to-sample distance (mm)	150	60
Detector-to-sample distance (mm)	100	100
X-ray optic lens	10×	20×
Scan range (º)	182	182
Number of raw projection images acquired over scan range	1000	1200
Physical size of reconstructed image pixel (μm)	1.60	0.50
Reconstructed image width and height	936 × 993	844 × 980

Supplementary Table S3 | Data matrix for cladistics analysis. This data matrix is built upon Wills et al.[8], Liu et al.[6], and Neuhaus[9].

	1	2	3	4	5	6	7	8	9	10	11	12	13	14	15	16	17	18	19	20	21	22	23	24	25	26	27
Eokinorhynchus_rarus	2	0	2	2	1	0	0	3	-	3	0	1	0	-	1	0	-	1	1	3	0	0	0	2	3	2	-
Eopriapulites_sphinx	?	0	2	?	1	2	1	3	-	3	0	0	2	1	1	0	-	1	?	3	-	1	0	2	0	0	-
Gastrotricha	-	0	0	0	-	-	-	-	-	-	-	-	0	-	-	-	-	-	-	0	-	-	-	-	-	-	-
Antygomonas	2	0	2	2	1	2	0	9	3	3	0	1	0	-	1	8	1	1	1	2	0	0	0	2	0	1	1
Campyloderes	2	0	2	2	1	2	0	9	3	3	0	1	0	-	1	8	5	1	1	2	0	0	0	2	0	1	1
Cateria	2	0	2	2	1	2	0	9	?	3	0	1	0	-	1	8	2	1	1	2	0	0	0	2	0	1	1
Centroderes	2	0	2	2	1	2	0	9	3	3	0	1	0	-	1	8	1	1	1	2	0	0	0	2	0	1	1
Cephalorhyncha	2	0	2	2	1	2	0	9	1	3	0	1	0	-	1	8	1	1	1	2	0	0	0	2	0	1	1
Condyloderes	2	0	2	2	1	2	0	9	3	3	0	1	0	-	1	8	0	1	1	2	0	0	0	2	0	1	1
Dracoderes	2	0	2	2	1	2	0	9	2	3	0	1	0	-	1	8	(02)	1	1	2	0	0	0	2	0	1	1
Echinoderes	2	0	2	2	1	2	0	9	1	3	0	1	0	-	1	8	1	1	1	2	0	0	0	2	0	1	1
Fissuroderes	2	0	2	2	1	2	0	9	1	3	0	1	0	-	1	8	1	1	1	2	0	0	0	2	0	1	1
Kinorhynchus	2	0	2	2	1	2	0	9	3	3	0	1	0	-	1	8	0	1	1	2	0	0	0	2	0	1	1
Meristoderes	2	0	2	2	1	2	0	9	1	3	0	1	0	-	1	8	1	1	1	2	0	0	0	2	0	1	1
Neocentrophyes	2	0	2	2	1	2	0	9	3	3	0	1	0	-	1	8	(02)	1	1	2	0	0	0	2	0	1	1
Paracentrophyes	2	0	2	2	1	2	0	9	3	3	0	1	0	-	1	8	(02)	1	1	2	0	0	0	2	0	1	1
Polacanthoderes	2	0	2	2	1	2	0	9	1	3	0	1	0	-	1	8	1	1	1	2	0	0	0	2	0	1	1
Pycnophyes	2	0	2	2	1	2	0	9	3	3	0	1	0	-	1	8	0	1	1	2	0	0	0	2	0	1	1
Semnoderes	2	0	2	2	1	2	0	9	3	3	0	1	0	-	1	8	1	1	1	2	0	0	0	2	0	1	1
Sphenoderes	2	0	2	2	1	2	0	9	3	3	0	1	0	-	1	8	1	1	1	2	0	0	0	2	0	1	1
Triodontoderes	2	0	2	2	1	2	0	9	3	3	0	1	0	-	1	8	0	1	1	2	0	0	0	2	0	1	1
Tubulideres	2	0	2	2	1	2	0	9	3	3	0	1	0	-	1	8	1	1	1	2	0	0	0	2	0	1	1
Wollunquaderes	2	0	2	2	1	2	0	9	3	3	0	1	0	-	1	8	1	1	1	2	0	0	0	2	0	1	1
Zelinkaderes	2	0	2	2	1	2	0	9	3	3	0	1	0	-	1	8	1	1	1	2	0	0	0	2	0	1	1
Loricifera	2	0	2	2	1	2	0	9	0	3	0	1	1	0	1	8	(34)	0	1	2	0	1	0	1	0	3	-
Nematomorpha	0	0	1	0	1	0	1	1	-	1	0	0	?	?	?	?	?	?	?	?	?	?	?	?	?	0	-
Nematoda	0	0	1	0	1	0	1	1	-	1	0	0	?	?	?	?	?	?	?	?	?	?	?	?	?	0	-
Aysheaia	-	1	0	0	-	-	-	-	-	-	-	-	-	-	-	-	-	-	-	-	-	-	-	-	-	-	-
Peripatus	-	0	0	0	-	-	-	-	-	-	-	-	-	-	-	-	-	-	-	-	-	-	-	-	-	-	-
Kerygmachela	-	1	0	0	-	-	-	-	-	-	-	-	-	-	-	-	-	-	-	-	-	-	-	-	-	-	-
Microdictyon	-	1	0	0	-	-	-	-	-	-	-	-	-	-	-	-	-	-	-	-	-	-	-	-	-	-	-
Tardigrada	-	1	0	0	-	-	-	-	-	-	-	-	-	-	-	-	-	-	-	-	-	-	-	-	-	-	-
Acanthopriapulus_horridus	2	0	2	1	1	1	0	3	-	2	0	0	?	?	?	?	-	?	?	?	?	?	0	2	?	0	-
Ancalagon_minor	0	0	(12)	?	1	2	0	2	-	1	0	0	0	-	1	3	-	0	0	2	0	0	0	2	0	0	-
Anningvermis_multispinosus	?	?	(12)	?	0	2	-	-	-	1	-	-	2	1	3	1	-	1	1	1	?	1	0	0	0	0	-
Chalazoscolex_pharkus	?	0	(12)	?	1	?	?	1	-	?	?	0	?	-	?	?	-	?	?	?	?	?	1	?	?	0	-
Corynetis_brevis	?	?	(12)	?	0	2	-	-	-	1	-	-	2	1	3	1	-	1	1	1	?	1	0	0	0	0	-
Cricocosmia_jinningensis	?	0	(12)	?	1	2	?	2	-	1	0	0	0	0	3	0	-	1	1	1	0	1	1	0	0	0	-
Fieldia_lanceolata	?	0	(12)	?	1	2	?	2	-	1	0	0	0	-	1	0	-	0	0	3	0	0	0	?	0	0	-
Guanduscolex_minor	?	0	(12)	?	1	2	0	?	-	1	0	0	0	0	3	0	-	1	1	3	0	0	0	0	0	0	-
Halicryptus_higginsi	2	0	2	1	1	1	0	5	-	2	0	0	2	0	3	1	-	2	1	1	2	1	0	2	1	0	-
Halicryptus_spinulosus	2	0	2	1	1	1	0	5	-	2	0	0	2	0	3	1	-	2	1	1	2	1	0	2	1	0	-
Laojieella_thecata	?	0	(12)	?	1	1	?	1	-	1	0	0	0	0	3	0	-	1	1	2	0	0	0	0	0	0	-
Louisella_pedunculata	1	0	(12)	?	1	2	0	0	-	1	0	0	2	1	3	0	-	1	1	3	0	1	1	0	0	0	-
Maccabeus_cirratus	2	0	2	1	1	1	0	(27)	-	2	0	0	1	0	2	5	-	2	1	1	(23)	0	0	2	0	0	-
Maccabeus_tentaculatus	2	0	2	1	1	1	0	(27)	-	2	0	0	1	0	2	5	-	2	1	1	(23)	0	0	2	0	0	-
Maotianshania_cylindrica	2	0	(12)	?	1	2	0	?	-	?	0	0	?	?	3	?	-	?	?	3	?	0	0	0	1	0	-
Markuelia	2	0	2	?	1	0	?	1	-	2	0	0	0	-	0	-	-	-	-	0	-	-	-	-	-	0	-
Meiopriapulus_fijiensis	2	0	2	1	1	1	0	8	-	3	0	0	1	0	2	6	-	3	3	3	0	0	0	1	0	0	-
Ottoia_prolifica	2	0	(12)	?	1	2	0	2	-	3	0	0	2	0	3	4	-	(12)	2	3	0	1	1	0	1	0	-
Palaeopriapulite_sparvus	?	0	2	?	1	1	0	2	-	1	0	0	?	?	?	?	-	?	?	?	?	?	?	?	?	0	-
Palaeoscolex_piscatorum	?	0	(12)	?	1	0	?	?	-	?	?	0	?	?	?	0	-	1	1	?	?	?	0	0	0	0	-
Paraselkirkia_jinningensis	1	0	(12)	?	1	0	0	1	-	(23)	0	0	2	0	2	?	-	?	?	3	0	0	0	0	0	0	-
Paratubiluchus_bicaudatus	?	0	(12)	1	1	1	0	?	-	2	0	0	0	0	(123)	0	-	1	1	?	?	0	0	?	?	0	-
Priapulites_konecniorum	2	0	(12)	?	1	1	0	3	-	1	1	0	?	?	?	?	-	?	?	?	?	?	0	2	?	0	-
Priapulopsis_australis	2	0	2	1	1	1	0	4	-	2	1	0	1	0	3	0	-	2	1	2	1	1	0	2	1	0	-
Priapulopsis_bicaudatus	2	0	2	1	1	1	0	4	-	2	1	0	1	0	3	2	-	2	1	2	1	1	0	2	1	0	-
Priapulopsis_cnidephorus	2	0	(12)	1	1	1	?	?	-	?	?	0	?	0	3	?	-	?	?	?	?	?	?	?	?	0	-
Priapulus_abyssorum	2	0	2	1	1	1	0	3	-	2	1	0	1	0	3	1	-	2	1	1	3	1	0	2	1	0	-
Priapulus_caudatus	2	0	2	1	1	1	0	3	-	2	1	0	1	0	3	1	-	2	1	1	3	1	0	2	1	0	-
Priapulus_tuberculatospinosus	2	0	2	1	1	1	0	3	-	2	1	0	1	0	3	1	-	2	1	1	3	1	0	2	1	0	-
Scolecofurca_rara	?	0	?	?	1	?	?	0	-	?	0	0	0	-	3	0	-	1	1	?	0	1	1	?	0	0	-
Selkirkia_columbia	1	0	(12)	?	1	0	0	1	-	3	0	0	2	0	2	1	-	2	2	3	0	0	0	0	1	0	-
Sicyophorus_rarus	?	0	2	?	1	1	0	2	-	1	0	0	?	0	?	0	-	1	1	2	?	?	1	?	0	0	-
Tabelliscolex_hexagonus	?	0	(12)	?	1	2	?	2	-	1	0	0	0	0	3	0	-	1	1	1	0	1	?	?	?	0	-
Tabelliscolex_maanshanensis	?	0	(12)	?	1	2	?	2	-	1	0	0	0	0	3	0	-	1	1	1	0	1	?	?	?	0	-
Tubiluchus_arcticus	2	0	2	1	1	1	0	6	-	2	0	0	1	0	2	7	-	3	3	3	0	0	0	1	0	1	-
Tubiluchus_australensis	2	0	2	1	1	1	0	6	-	2	0	0	1	0	2	7	-	3	3	3	0	0	0	1	0	0	-
Tubiluchus_corallicola	2	0	2	1	1	1	0	6	-	2	0	0	1	0	2	7	-	3	3	3	0	0	0	1	0	0	-
Tubiluchus_philippinensis	2	0	2	1	1	1	0	6	-	2	0	0	1	0	2	7	-	3	3	3	0	0	0	1	0	0	-
Tubiluchus_remanei	2	0	2	1	1	1	0	6	-	2	0	0	1	0	2	7	-	3	3	3	0	0	0	1	0	0	-
Tubiluchus_troglodytes	2	0	2	1	1	1	0	6	-	2	0	0	1	0	2	7	-	3	3	3	0	0	0	1	0	0	-
Tubiluchus_vanuatensis	2	0	2	1	1	1	0	6	-	2	0	0	1	0	2	7	-	3	3	3	0	0	0	0	0	1	-
Tylotites_petiolaris	?	0	(12)	?	1	2	?	2	-	1	0	0	0	0	2	0	-	1	1	2	0	0	1	0	0	0	-
Xiaoheiqingella_peculiaris	?	0	2	?	1	1	0	1	-	2	0	0	1	0	(123)	0	-	1	1	?	?	?	?	?	?	0	-
Xystoscolex_boreogyrus	?	0	(12)	1	1	0	?	1	-	?	?	0	?	-	?	0	-	1	1	?	?	?	?	0	0	0	-
Yunnanpriapulus_halteroformis	?	0	2	?	1	1	0	?	-	2	0	0	?	?	(123)	?	-	?	?	?	?	?	?	?	?	0	-

Supplementary Table S3 | Dat

	28	29	30	31	32	33	34	35	36	37	38	39	40	41	42	43	44	45	46	47	48	49	50	51	52	53	54
Eokinorhynchus_rarus	-	-	-	-	0	0	0	2	2	1	1	0	0	0	0	0	0	1	1	1	0	1	0	0	0	0	0
Eopriapulites_sphinx	-	-	-	-	?	0	0	1	3	0	-	-	-	-	-	-	-	0	-	-	-	-	-	-	-	-	0
Gastrotricha	-	-	-	-	0	0	0	0	0	0	-	-	-	-	-	-	-	1	-	-	-	-	-	-	-	-	0
Antygomonas	0	0	0	5	0	0	0	2	1	1	0	1	3	1	1	1	1	1	1	0	1	0	1	1	1	0	0
Campyloderes	0	0	0	4	0	0	0	2	1	1	0	0	3	1	1	1	1	1	0	0	1	0	1	1	1	0	0
Cateria	1	0	0	?	0	0	0	2	1	1	0	2	2	2	2	2	2	1	0	0	1	0	1	1	1	0	0
Centroderes	0	0	0	5	0	0	0	2	1	1	0	0	3	1	1	1	1	1	0	0	1	0	1	1	1	0	0
Cephalorhyncha	0	0	0	5	0	0	0	2	1	1	0	0	2	1	1	1	1	1	0	0	1	0	1	1	0	0	0
Condyloderes	0	0	1	5	0	0	0	2	1	1	0	0	3	1	1	1	1	1	1	1	1	0	1	0	1	0	0
Dracoderes	0	0	0	3	0	0	0	2	1	1	0	0	3	1	1	1	1	1	0	0	1	0	1	0	0	0	0
Echinoderes	0	0	0	5	0	0	0	2	1	1	0	0	0	1	1	1	1	1	0	0	1	0	1	(01)	0	0	0
Fissuroderes	0	0	0	5	0	0	0	2	1	1	0	0	3	1	1	1	1	1	0	0	1	0	1	1	0	0	0
Kinorhynchus	0	0	0	(02)	0	0	0	2	1	1	0	3	3	1	1	1	1	1	0	0	0	0	0	0	0	0	0
Meristoderes	0	0	0	5	0	0	0	2	1	1	0	0	0	1	1	1	1	1	0	0	1	0	1	1	0	0	0
Neocentrophyes	0	0	0	1	0	0	0	2	1	1	0	2	3	1	1	1	1	1	0	0	0	0	0	0	0	1	0
Paracentrophyes	0	0	0	1	0	0	0	2	1	1	0	2	3	1	1	1	1	1	0	0	0	0	1	0	0	1	0
Polacanthoderes	0	0	0	5	0	0	0	2	1	1	0	0	3	1	1	1	1	1	0	0	1	1	1	1	0	0	0
Pycnophyes	0	0	0	(02)	0	0	0	2	1	1	0	3	3	1	1	1	1	1	0	0	0	0	1	0	0	0	0
Semnoderes	0	0	0	5	0	0	0	2	1	1	0	5	3	1	1	1	1	1	1	0	1	0	1	1	1	0	0
Sphenoderes	0	0	0	5	0	0	0	2	1	1	0	4	3	1	1	1	1	1	1	0	1	0	1	1	1	0	0
Triodontoderes	1	1	0	4	0	0	0	2	1	1	0	0	3	1	3	3	2	1	1	0	1	0	1	1	1	0	0
Tubulideres	(01)	0	0	5	0	0	0	2	1	1	0	0	1	1	1	1	1	1	0	0	1	0	1	1	1	0	0
Wollunquaderes	0	0	0	5	0	0	0	2	1	1	0	0	3	1	1	1	1	1	1	1	1	0	1	1	1	0	0
Zelinkaderes	1	1	0	5	0	1	0	2	1	1	0	0	0	3	3	3	2	1	1	0	1	0	1	1	1	0	0
Loricifera	-	-	-	-	0	0	0	0	0	0	-	-	-	-	-	-	-	0	-	-	-	-	-	-	-	-	0
Nematomorpha	-	-	-	-	2	0	0	(01)	5	0	-	-	-	-	-	-	-	1	-	-	-	-	-	-	-	-	1
Nematoda	-	-	-	-	2	0	0	(01)	5	0	-	-	-	-	-	-	-	1	-	-	-	-	-	-	-	-	0
Aysheaia	-	-	-	-	1	0	1	2	3	0	-	-	-	-	-	-	-	1	-	-	-	-	-	-	-	-	1
Peripatus	-	-	-	-	1	0	1	2	?	0	-	-	-	-	-	-	-	1	-	-	-	-	-	-	-	-	1
Kerygmachela	-	-	-	-	1	0	1	2	5	1	0	0	0	0	0	0	0	1	0	0	0	0	1	0	0	0	1
Microdictyon	-	-	-	-	1	0	1	2	4	1	0	0	0	0	0	0	0	0	0	0	0	0	0	0	0	0	0
Tardigrada	-	-	-	-	0	0	1	2	0	1	0	0	0	0	-	-	-	1	1	0	1	1	0	0	0	0	0
Acanthopriapulus_horridus	-	-	-	-	0	0	0	1	3	0	-	-	-	-	-	-	-	0	-	-	-	-	-	-	-	-	1
Ancalagon_minor	-	-	-	-	1	0	0	1	5	0	-	-	-	-	-	-	-	1	-	-	-	-	-	-	-	-	0
Anningvermis_multispinosus	-	-	-	-	1	0	0	1	3	0	-	-	-	-	-	-	-	1	-	-	-	-	-	-	-	-	1
Chalazoscolex_pharkus	-	-	-	-	1	0	0	1	5	0	-	-	-	-	-	-	-	0	-	-	-	-	-	-	-	-	0
Corynetis_brevis	-	-	-	-	1	0	0	1	3	0	-	-	-	-	-	-	-	1	-	-	-	-	-	-	-	-	1
Cricocosmia_jinningensis	-	-	-	-	2	0	0	1	4	0	-	-	-	-	-	-	-	0	-	-	-	-	-	-	-	-	0
Fieldia_lanceolata	-	-	-	-	1	0	0	0	0	0	-	-	-	-	-	-	-	1	-	-	-	-	-	-	-	-	0
Guanduscolex_minor	-	-	-	-	1	0	0	1	4	0	-	-	-	-	-	-	-	0	-	-	-	-	-	-	-	-	0
Halicryptus_higginsi	-	-	-	-	1	0	0	1	5	0	-	-	-	-	-	-	-	1	-	-	-	-	-	-	-	-	0
Halicryptus_spinulosus	-	-	-	-	0	0	0	1	4	0	-	-	-	-	-	-	-	1	-	-	-	-	-	-	-	-	0
Laojieella_thecata	-	-	-	-	1	0	0	0	0	0	-	-	-	-	-	-	-	0	-	-	-	-	-	-	-	-	0
Louisella_pedunculata	-	-	-	-	1	0	0	1	?	0	-	-	-	-	-	-	-	1	-	-	-	-	-	-	-	-	2
Maccabeus_cirratus	-	-	-	-	0	0	0	1	4	0	-	-	-	-	-	-	-	0	-	-	-	-	-	-	-	-	0
Maccabeus_tentaculatus	-	-	-	-	0	0	0	1	4	0	-	-	-	-	-	-	-	0	-	-	-	-	-	-	-	-	0
Maotianshania_cylindrica	-	-	-	-	2	0	0	1	4	0	-	-	-	-	-	-	-	0	-	-	-	-	-	-	-	-	0
Markuelia	-	-	-	-	1	0	0	1	4	0	-	-	-	-	-	-	-	(01)	-	-	-	-	-	-	-	-	0
Meiopriapulus_fijiensis	-	-	-	-	0	0	0	0	0	0	-	-	-	-	-	-	-	1	-	-	-	-	-	-	-	-	0
Ottoia_prolifica	-	-	-	-	0	0	0	1	4	0	-	-	-	-	-	-	-	0	-	-	-	-	-	-	-	-	0
Palaeopriapulite_sparvus	-	-	-	-	0	0	0	0	0	0	-	-	-	-	-	-	-	0	-	-	-	-	-	-	-	-	0
Palaeoscolex_piscatorum	-	-	-	-	2	0	0	1	5	0	-	-	-	-	-	-	-	0	-	-	-	-	-	-	-	-	0
Paraselkirkia_jinningensis	-	-	-	-	1	0	0	0	0	0	-	-	-	-	-	-	-	0	-	-	-	-	-	-	-	-	?
Paratubiluchus_bicaudatus	-	-	-	-	0	0	0	0	0	0	-	-	-	-	-	-	-	1	-	-	-	-	-	-	-	-	0
Priapulites_konecniorum	-	-	-	-	0	0	0	1	3	0	-	-	-	-	-	-	-	0	-	-	-	-	-	-	-	-	0
Priapulopsis_australis	-	-	-	-	0	0	0	1	3	0	-	-	-	-	-	-	-	0	-	-	-	-	-	-	-	-	1
Priapulopsis_bicaudatus	-	-	-	-	0	0	0	1	3	0	-	-	-	-	-	-	-	0	-	-	-	-	-	-	-	-	1
Priapulopsis_cnidephorus	-	-	-	-	0	0	0	1	3	0	-	-	-	-	-	-	-	0	-	-	-	-	-	-	-	-	1
Priapulus_abyssorum	-	-	-	-	0	0	0	1	3	0	-	-	-	-	-	-	-	0	-	-	-	-	-	-	-	-	1
Priapulus_caudatus	-	-	-	-	0	0	0	1	3	0	-	-	-	-	-	-	-	0	-	-	-	-	-	-	-	-	1
Priapulus_tuberculatospinosus	-	-	-	-	0	0	0	1	3	0	-	-	-	-	-	-	-	0	-	-	-	-	-	-	-	-	1
Scolecofurca_rara	-	-	-	-	?	0	0	1	?	0	-	-	-	-	-	-	-	0	-	-	-	-	-	-	-	-	0
Selkirkia_columbia	-	-	-	-	1	0	0	0	0	0	-	-	-	-	-	-	-	0	-	-	-	-	-	-	-	-	1
Sicyophorus_rarus	-	-	-	-	0	0	0	0	0	0	-	-	-	-	-	-	-	0	-	-	-	-	-	-	-	-	0
Tabelliscolex_hexagonus	-	-	-	-	2	0	0	1	4	0	-	-	-	-	-	-	-	0	-	-	-	-	-	-	-	-	0
Tabelliscolex_maanshanensis	-	-	-	-	2	0	0	1	4	0	-	-	-	-	-	-	-	0	-	-	-	-	-	-	-	-	0
Tubiluchus_arcticus	-	-	-	-	0	0	0	0	0	0	-	-	-	-	-	-	-	1	-	-	-	-	-	-	-	-	0
Tubiluchus_australensis	-	-	-	-	0	0	0	0	0	0	-	-	-	-	-	-	-	1	-	-	-	-	-	-	-	-	0
Tubiluchus_corallicola	-	-	-	-	0	0	0	0	0	0	-	-	-	-	-	-	-	1	-	-	-	-	-	-	-	-	0
Tubiluchus_philippinensis	-	-	-	-	0	0	0	0	0	0	-	-	-	-	-	-	-	1	-	-	-	-	-	-	-	-	0
Tubiluchus_remanei	-	-	-	-	0	0	0	0	0	0	-	-	-	-	-	-	-	1	-	-	-	-	-	-	-	-	0
Tubiluchus_troglodytes	-	-	-	-	0	0	0	0	0	0	-	-	-	-	-	-	-	1	-	-	-	-	-	-	-	-	0
Tubiluchus_vanuatensis	-	-	-	-	0	0	0	0	0	0	-	-	-	-	-	-	-	1	-	-	-	-	-	-	-	-	0
Tylotites_petiolaris	-	-	-	-	1	0	0	1	3	0	-	-	-	-	-	-	-	1	-	-	-	-	-	-	-	-	0
Xiaoheiqingella_peculiaris	-	-	-	-	1	0	0	1	4	0	-	-	-	-	-	-	-	0	-	-	-	-	-	-	-	-	?
Xystoscolex_boreogyrus	-	-	-	-	1	0	0	1	5	0	-	-	-	-	-	-	-	0	-	-	-	-	-	-	-	-	0
Yunnanpriapulus_halteroformis	-	-	-	-	1	0	0	1	?	0	-	-	-	-	-	-	-	0	-	-	-	-	-	-	-	-	1

Supplementary Table S3 | Dat

	55	56	57	58	59	60	61	62	63	64	65	66	67	68	69	70	71	72	73	74	75	76	77	78	79	80	81
Eokinorhynchus_rarus	2	3	0	0	0	?	0	0	0	0	0	0	0	0	0	-	-	-	0	0	?	?	?	?	?	?	?
Eopriapulites_sphinx	0	0	0	0	0	0	?	0	?	?	?	?	?	?	?	?	?	?	?	?	?	?	?	?	?	?	?
Gastrotricha	0	0	0	0	0	0	0	0	0	0	0	0	0	0	0	-	-	-	0	0	0	0	0	0	0	0	0
Antygomonas	0	0	0	0	0	0	0	0	0	0	0	0	0	0	0	-	-	-	0	0	0	?	?	0	1	1	1
Campyloderes	0	0	0	0	0	0	0	0	0	0	0	0	0	0	0	-	-	-	0	0	0	?	?	0	1	1	1
Cateria	0	0	0	0	0	0	0	0	0	0	0	0	0	0	0	-	-	-	0	0	0	?	?	0	1	1	1
Centroderes	0	0	0	0	0	0	0	0	0	0	0	0	0	0	0	-	-	-	0	0	0	?	?	0	1	1	1
Cephalorhyncha	0	0	0	0	0	0	0	0	0	0	0	0	0	0	0	-	-	-	0	0	0	?	?	0	1	1	1
Condyloderes	0	0	0	0	0	0	0	0	0	0	0	0	0	0	0	-	-	-	0	0	0	?	?	0	1	1	1
Dracoderes	0	0	0	0	0	0	0	0	0	0	0	0	0	0	0	-	-	-	0	0	0	?	?	0	1	1	1
Echinoderes	0	0	0	0	0	0	0	0	0	0	0	0	0	0	0	-	-	-	0	0	0	?	?	0	1	1	1
Fissuroderes	0	0	0	0	0	0	0	0	0	0	0	0	0	0	0	-	-	-	0	0	0	?	?	0	1	1	1
Kinorhynchus	0	0	0	0	0	0	0	0	0	0	0	0	0	0	0	-	-	-	0	0	0	?	?	0	1	1	1
Meristoderes	0	0	0	0	0	0	0	0	0	0	0	0	0	0	0	-	-	-	0	0	0	?	?	0	1	1	1
Neocentrophyes	0	0	0	0	0	0	0	0	0	0	0	0	0	0	0	-	-	-	0	0	0	?	?	0	1	1	1
Paracentrophyes	0	0	0	0	0	0	0	0	0	0	0	0	0	0	0	-	-	-	0	0	0	?	?	0	1	1	1
Polacanthoderes	0	0	0	0	0	0	0	0	0	0	0	0	0	0	0	-	-	-	0	0	0	?	?	0	1	1	1
Pycnophyes	0	0	0	0	0	0	0	0	0	0	0	0	0	0	0	-	-	-	0	0	0	?	?	0	1	1	1
Semnoderes	0	0	0	0	0	0	0	0	0	0	0	0	0	0	0	-	-	-	0	0	0	?	?	0	1	1	1
Sphenoderes	0	0	0	0	0	0	0	0	0	0	0	0	0	0	0	-	-	-	0	0	0	?	?	0	1	1	1
Triodontoderes	0	0	0	0	0	0	0	0	0	0	0	0	0	0	0	-	-	-	0	0	0	?	?	0	1	1	1
Tubulideres	0	0	0	0	0	0	0	0	0	0	0	0	0	0	0	-	-	-	0	0	0	?	?	0	1	1	1
Wollunquaderes	0	0	0	0	0	0	0	0	0	0	0	0	0	0	0	-	-	-	0	0	0	?	?	0	1	1	1
Zelinkaderes	0	0	0	0	0	0	0	0	0	0	0	0	0	0	0	-	-	-	0	0	0	?	?	0	1	1	1
Loricifera	0	0	0	0	0	1	0	0	0	0	0	0	0	0	0	-	-	-	0	0	0	0	0	0	1	1	1
Nematomorpha	0	0	0	0	0	0	0	0	1	0	0	0	1	0	0	-	-	-	1	0	0	0	1	1	1	1	1
Nematoda	0	0	0	0	0	0	0	0	0	0	(01)	0	0	0	0	-	-	-	0	0	0	0	1	1	1	1	1
Aysheaia	0	0	0	0	0	0	0	0	0	0	0	0	0	?	0	-	-	-	0	0	0	?	?	?	?	?	?
Peripatus	0	0	0	0	0	0	0	0	0	0	0	0	1	0	0	-	-	-	0	0	0	0	0	?	0	0	0
Kerygmachela	0	0	0	0	0	0	0	0	0	0	0	0	?	0	0	-	-	?	0	0	0	?	?	?	?	?	?
Microdictyon	2	1	0	0	0	0	0	0	0	0	0	0	0	0	0	-	-	-	0	0	0	?	?	?	?	?	?
Tardigrada	(01)	1	0	0	0	0	0	0	0	0	0	0	1	0	0	-	-	-	0	0	0	0	0	?	1	0	0
Acanthopriapulus_horridus	0	0	0	0	0	0	0	0	0	1	0	0	1	0	1	1	1	1	0	0	0	1	0	0	1	1	1
Ancalagon_minor	0	0	0	0	0	0	0	0	0	0	0	0	0	0	0	-	-	-	0	0	0	?	?	?	?	?	?
Anningvermis_multispinosus	0	0	0	0	0	0	0	0	0	0	?	0	?	?	1	0	0	0	0	0	0	?	?	?	?	?	?
Chalazoscolex_pharkus	2	2	0	0	0	0	0	0	0	0	1	0	0	0	0	-	-	-	0	0	?	?	?	?	?	?	?
Corynetis_brevis	0	0	0	0	0	0	0	0	0	1	?	0	0	?	1	0	0	0	0	0	0	?	?	?	?	?	?
Cricocosmia_jinningensis	2	1	0	1	0	0	0	0	0	0	0	0	0	0	0	-	-	-	1	0	0	?	?	?	?	?	?
Fieldia_lanceolata	0	0	0	0	0	0	0	0	0	0	0	0	0	0	0	-	-	-	0	0	0	?	?	?	?	?	?
Guanduscolex_minor	1	1	0	0	0	0	0	0	0	0	0	0	0	0	0	-	-	-	0	0	0	?	?	?	?	?	?
Halicryptus_higginsi	0	0	0	0	0	0	0	0	0	1	0	0	1	1	0	-	-	-	0	1	0	1	0	0	1	1	1
Halicryptus_spinulosus	0	0	0	0	0	0	0	0	0	1	0	0	1	1	0	-	-	-	0	1	0	1	0	0	1	1	1
Laojieella_thecata	0	0	0	0	0	0	0	0	0	0	0	0	0	0	1	0	2	0	?	0	0	?	?	?	?	?	?
Louisella_pedunculata	?	?	0	0	0	0	0	0	0	0	1	0	0	0	0	-	-	-	0	0	0	?	?	?	?	?	?
Maccabeus_cirratus	0	0	0	0	1	1	0	1	1	0	0	1	1	1	0	-	-	-	0	0	0	1	0	0	1	1	1
Maccabeus_tentaculatus	0	0	0	0	1	1	0	1	1	0	0	1	1	1	0	-	-	-	0	0	0	1	0	0	1	1	1
Maotianshania_cylindrica	0	0	0	0	0	0	0	0	0	0	0	0	0	0	0	-	-	-	1	0	0	?	?	?	?	?	?
Markuelia	0	0	0	0	0	0	0	0	0	0	0	0	0	0	0	-	-	-	1	0	0	?	?	?	?	?	?
Meiopriapulus_fijiensis	0	0	1	1	0	2	0	0	1	0	0	0	0	0	0	-	-	-	0	0	1	1	0	0	1	1	0
Ottoia_prolifica	0	0	0	0	0	0	0	0	1	0	1	0	0	0	0	-	-	-	0	0	0	1	0	?	?	?	?
Palaeopriapulite_sparvus	0	0	0	0	0	0	0	0	0	0	0	0	0	0	0	-	-	-	0	0	0	?	?	?	?	?	?
Palaeoscolex_piscatorum	2	2	0	0	0	0	0	0	0	0	0	0	0	0	0	-	-	-	1	0	0	?	?	?	?	?	?
Paraselkirkia_jinningensis	0	0	0	0	0	0	0	2	0	0	0	0	0	0	0	-	-	-	?	0	0	?	?	?	?	?	?
Paratubiluchus_bicaudatus	0	0	1	?	0	?	?	0	?	?	?	0	?	?	1	0	2	0	0	0	1	?	?	?	?	?	?
Priapulites_konecniorum	0	0	0	0	0	0	0	0	0	0	0	0	1	0	1	0	1	0	0	0	0	?	?	?	?	?	?
Priapulopsis_australis	0	0	1	0	0	0	0	0	0	1	0	0	1	0	1	0	0	1	0	0	0	1	0	0	1	1	1
Priapulopsis_bicaudatus	0	0	1	0	0	0	0	0	0	1	0	0	1	0	1	0	2	1	0	0	0	1	0	0	1	1	1
Priapulopsis_cnidephorus	0	0	1	0	0	0	0	0	0	?	0	0	1	0	1	?	?	?	0	0	?	1	0	0	?	?	?
Priapulus_abyssorum	0	0	0	0	0	0	0	0	0	1	0	0	1	0	1	1	1	1	0	0	0	1	0	0	1	1	1
Priapulus_caudatus	0	0	0	0	0	0	0	0	0	1	0	0	1	0	1	1	1	1	0	2	0	1	0	0	1	1	1
Priapulus_tuberculatospinosus	0	0	0	0	0	0	0	0	0	1	0	0	1	0	1	1	1	2	0	2	0	1	0	0	1	1	1
Scolecofurca_rara	0	0	0	0	0	0	0	0	0	0	?	0	0	0	0	-	-	-	?	0	0	?	?	?	?	?	?
Selkirkia_columbia	0	0	0	0	0	0	0	2	0	0	0	0	0	0	0	-	-	-	0	0	0	?	?	?	?	?	?
Sicyophorus_rarus	0	0	0	0	0	0	0	0	0	0	0	0	0	0	0	-	-	-	0	0	0	?	?	?	?	?	?
Tabelliscolex_hexagonus	2	1	0	1	0	0	0	0	0	0	0	0	0	0	0	-	-	-	1	0	0	?	?	?	?	?	?
Tabelliscolex_maanshanensis	2	1	0	1	0	0	0	0	0	0	0	0	0	0	0	-	-	-	1	0	0	?	?	?	?	?	?
Tubiluchus_arcticus	0	0	1	1	0	1	1	0	0	0	0	0	1	0	2	0	0	0	0	0	1	1	0	0	1	1	0
Tubiluchus_australensis	0	0	1	1	0	1	1	0	0	0	0	0	1	0	1	0	0	0	0	0	1	1	0	0	1	1	0
Tubiluchus_corallicola	0	0	1	1	0	1	1	0	0	0	0	0	1	0	1	0	0	0	0	0	1	1	0	0	1	1	0
Tubiluchus_philippinensis	0	0	1	1	0	1	1	0	0	0	0	0	1	0	1	0	0	0	0	0	1	1	0	0	1	1	0
Tubiluchus_remanei	0	0	1	1	0	1	1	0	0	0	0	0	1	0	2	0	0	0	0	0	1	1	0	0	1	1	0
Tubiluchus_troglodytes	0	0	1	1	0	2	1	0	0	0	0	0	1	0	2	0	0	0	0	0	1	1	0	0	1	1	0
Tubiluchus_vanuatensis	0	0	1	1	0	1	1	0	0	0	0	0	1	0	1	0	0	0	0	0	1	1	0	0	1	1	0
Tylotites_petiolaris	2	2	0	?	0	0	0	0	0	0	0	0	0	0	0	-	-	-	1	0	0	?	?	?	?	?	?
Xiaoheiqingella_peculiaris	0	0	0	0	0	0	0	0	0	1	?	0	0	?	1	0	2	0	0	0	0	?	?	?	?	?	?
Xystoscolex_boreogyrus	1	?	0	0	0	0	0	0	0	0	0	0	?	0	0	-	-	-	0	0	?	?	?	?	?	?	?
Yunnanpriapulus_halteroformis	0	0	0	0	0	0	0	0	0	1	?	0	0	?	1	0	0	0	0	0	0	?	?	?	?	?	?

Supplementary Table S3 | Dat

	82	83	84	85	86	87	88	89	90	91	92	93	94	95	96	97	98	99	100	101	102	103	104	105	106	107	108
Eokinorhynchus_rarus	?	?	0	0	0	1	1	?	0	1	?	?	?	?	?	?	?	?	?	?	?	?	?	?	1	1	1
Eopriapulites_sphinx	?	?	0	0	-	0	-	-	-	-	?	?	?	?	?	?	?	?	?	?	?	?	?	?	0	1	1
Gastrotricha	0	-	0	0	-	-	-	0	-	0	0	1	0	1	0	0	0	0	0	0	0	-	0	0	0	0	1
Antygomonas	0	1	0	0	-	1	0	0	-	1	0	1	0	1	0	0	0	0	1	1	1	0	0	0	0	1	1
Campyloderes	0	1	0	0	-	1	0	0	-	1	0	1	0	1	0	0	0	0	1	1	1	0	0	0	0	1	1
Cateria	0	1	0	0	-	1	0	0	-	1	0	1	0	1	0	0	0	0	1	1	1	0	0	0	0	1	1
Centroderes	0	1	0	0	-	1	0	0	-	1	0	1	0	1	0	0	0	0	1	1	1	0	0	0	0	1	1
Cephalorhyncha	0	1	0	0	-	1	0	0	-	1	0	1	0	1	0	0	0	0	1	1	1	0	0	0	0	1	1
Condyloderes	0	1	0	0	-	1	0	0	-	1	0	1	0	1	0	0	0	0	1	1	1	0	0	0	0	1	1
Dracoderes	0	1	0	0	-	1	0	0	-	1	0	1	0	1	0	0	0	0	1	1	1	0	0	0	0	1	1
Echinoderes	0	1	0	0	-	1	0	0	-	1	0	1	0	1	0	0	0	0	1	1	1	0	0	0	0	1	1
Fissuroderes	0	1	0	0	-	1	0	0	-	1	0	1	0	1	0	0	0	0	1	1	1	0	0	0	0	1	1
Kinorhynchus	0	1	0	0	-	1	0	0	-	1	0	1	0	1	0	0	0	0	1	1	1	0	0	0	0	1	1
Meristoderes	0	1	0	0	-	1	0	0	-	1	0	1	0	1	0	0	0	0	1	1	1	0	0	0	0	1	1
Neocentrophyes	0	1	0	0	-	1	0	0	-	1	0	1	0	1	0	0	0	0	1	1	1	0	0	0	0	1	1
Paracentrophyes	0	1	0	0	-	1	0	0	-	1	0	1	0	1	0	0	0	0	1	1	1	0	0	0	0	1	1
Polacanthoderes	0	1	0	0	-	1	0	0	-	1	0	1	0	1	0	0	0	0	1	1	1	0	0	0	0	1	1
Pycnophyes	0	1	0	0	-	1	0	0	-	1	0	1	0	1	0	0	0	0	1	1	1	0	0	0	0	1	1
Semnoderes	0	1	0	0	-	1	0	0	-	1	0	1	0	1	0	0	0	0	1	1	1	0	0	0	0	1	1
Sphenoderes	0	1	0	0	-	1	0	0	-	1	0	1	0	1	0	0	0	0	1	1	1	0	0	0	0	1	1
Triodontoderes	0	1	0	0	-	1	0	0	-	1	0	1	0	1	0	0	0	0	1	1	1	0	0	0	0	1	1
Tubulideres	0	1	0	0	-	1	0	0	-	1	0	1	0	1	0	0	0	0	1	1	1	0	0	0	0	1	1
Wollunquaderes	0	1	0	0	-	1	0	0	-	1	0	1	0	1	0	0	0	0	1	1	1	0	0	0	0	1	1
Zelinkaderes	0	1	0	0	-	1	0	0	-	1	0	1	0	1	0	0	0	0	1	1	1	0	0	0	0	1	1
Loricifera	0	1	1	1	0	-	-	0	0	1	0	2	1	1	0	0	0	0	2	1	1	0	0	0	0	1	1
Nematomorpha	0	0	1	0	-	0	-	0	0	1	1	0	0	0	0	0	0	1	2	1	0	-	1	(123)	1	0	1
Nematoda	0	0	0	0	-	0	-	0	-	0	1	0	0	0	0	0	0	1	2	(01)	1	(01)	(01)	2	0	0	1
Aysheaia	?	?	?	?	?	0	-	?	-	0	?	?	?	?	?	?	?	?	?	?	?	?	?	?	?	0	1
Peripatus	0	0	0	0	-	0	-	0	-	0	0	0	0	1	0	0	0	0	2	1	1	0	0	0	0	0	0
Kerygmachela	?	?	?	?	?	0	-	?	-	0	?	?	?	?	?	?	?	?	?	?	?	?	?	?	?	0	1
Microdictyon	?	?	?	?	?	0	-	?	-	0	?	?	?	?	?	?	?	?	?	?	?	?	?	?	?	0	1
Tardigrada	0	?	0	0	-	(01)	(-0)	0	-	0	(01)	0	0	1	0	0	0	0	2	(01)	1	(01)	0	0	?	0	(01)
Acanthopriapulus_horridus	1	1	1	1	1	0	-	1	1	1	0	2	1	1	0	0	0	0	?	?	?	?	?	0	0	1	1
Ancalagon_minor	?	?	?	?	?	0	-	?	?	?	?	?	?	?	?	?	?	?	?	?	?	?	?	?	?	1	1
Anningvermis_multispinosus	?	?	?	?	?	0	-	?	?	?	?	?	?	?	?	?	?	?	?	?	?	?	?	?	?	1	1
Chalazoscolex_pharkus	?	?	?	?	?	0	-	?	?	?	?	?	?	?	?	?	?	?	?	?	?	?	?	?	?	1	1
Corynetis_brevis	?	?	?	?	?	0	-	?	?	?	?	?	?	?	?	?	?	?	?	?	?	?	?	?	?	1	1
Cricocosmia_jinningensis	?	?	?	?	?	0	-	?	?	?	?	?	?	?	?	?	?	?	?	?	?	?	?	?	?	1	1
Fieldia_lanceolata	?	?	?	?	?	-	-	?	?	?	?	?	?	?	?	?	?	?	?	?	?	?	?	?	?	1	1
Guanduscolex_minor	?	?	?	?	?	0	-	?	?	?	?	?	?	?	?	?	?	?	?	?	?	?	?	?	?	1	1
Halicryptus_higginsi	1	1	1	1	1	0	-	1	0	1	0	2	1	1	0	0	0	0	2	1	1	(01)	0	0	0	1	1
Halicryptus_spinulosus	1	1	1	1	1	0	-	1	0	1	0	2	1	1	0	0	0	0	2	1	1	(01)	0	0	0	1	1
Laojieella_thecata	?	?	?	?	?	-	-	?	?	?	?	?	?	?	?	?	?	?	?	?	?	?	?	?	?	1	1
Louisella_pedunculata	?	?	?	?	?	0	-	?	?	?	?	?	?	?	?	?	?	?	?	?	?	?	?	?	?	1	1
Maccabeus_cirratus	1	1	1	1	1	0	-	1	0	1	0	2	1	1	0	0	0	0	?	?	?	?	?	0	0	1	1
Maccabeus_tentaculatus	1	1	1	1	1	0	-	1	0	1	0	2	1	1	0	0	0	0	?	?	?	?	?	0	0	1	1
Maotianshania_cylindrica	?	?	?	?	?	0	-	?	?	?	?	?	?	?	?	?	?	?	?	?	?	?	?	?	?	1	1
Markuelia	?	?	0	0	-	0	-	?	0	1	?	?	?	?	?	?	?	?	?	?	?	?	?	?	0	1	1
Meiopriapulus_fijiensis	1	1	0	0	-	-	-	-	-	?	0	2	1	1	0	0	0	0	?	?	?	?	1	0	0	1	1
Ottoia_prolifica	?	?	?	?	?	0	-	?	?	?	?	?	?	?	?	?	?	?	?	?	?	?	?	?	?	1	1
Palaeopriapulite_sparvus	?	?	?	1	0	-	-	?	?	?	?	?	?	?	?	?	?	?	?	?	?	?	?	?	?	1	1
Palaeoscolex_piscatorum	?	?	?	?	?	0	-	?	?	?	?	?	?	?	?	?	?	1	?	?	?	?	1	2	1	1	1
Paraselkirkia_jinningensis	?	?	?	?	?	-	-	?	?	?	?	?	?	?	?	?	?	?	?	?	?	?	?	?	?	1	1
Paratubiluchus_bicaudatus	?	?	?	?	?	-	-	?	?	?	?	?	?	?	?	?	?	?	?	?	?	?	?	?	?	1	1
Priapulites_konecniorum	?	?	?	?	?	0	-	?	?	?	?	?	?	?	?	?	?	?	?	?	?	?	?	?	?	1	1
Priapulopsis_australis	1	1	1	1	1	0	-	1	1	1	0	2	1	1	0	0	0	0	?	?	?	?	?	?	0	1	1
Priapulopsis_bicaudatus	1	1	1	1	1	0	-	1	1	1	0	2	1	1	0	0	0	0	?	?	?	?	?	?	0	1	1
Priapulopsis_cnidephorus	?	?	?	?	?	0	-	?	?	1	?	?	?	?	?	?	?	?	?	?	?	?	?	?	0	1	1
Priapulus_abyssorum	1	1	1	1	1	0	-	1	1	1	0	2	1	1	0	0	0	0	1	1	1	(01)	0	0	0	1	1
Priapulus_caudatus	1	1	1	1	1	0	-	1	1	1	0	2	1	1	0	0	0	0	1	1	1	(01)	0	0	0	1	1
Priapulus_tuberculatospinosus	1	1	1	1	1	0	-	1	1	1	0	2	1	1	0	0	0	0	1	1	1	(01)	0	0	0	1	1
Scolecofurca_rara	?	?	?	?	?	0	-	?	?	?	?	?	?	?	?	?	?	?	?	?	?	?	?	?	?	1	1
Selkirkia_columbia	?	?	?	?	0	-	-	?	?	?	?	?	?	?	?	?	?	?	?	?	?	?	?	?	?	1	1
Sicyophorus_rarus	?	?	?	1	0	-	-	?	?	?	?	?	?	?	?	?	?	?	?	?	?	?	?	?	?	1	1
Tabelliscolex_hexagonus	?	?	?	?	?	0	-	?	?	?	?	?	?	?	?	?	?	?	?	?	?	?	?	?	?	1	1
Tabelliscolex_maanshanensis	?	?	?	?	?	0	-	?	?	?	?	?	?	?	?	?	?	?	?	?	?	?	?	?	?	1	1
Tubiluchus_arcticus	1	1	1	1	0	-	-	1	0	1	0	2	1	1	0	0	0	0	?	?	?	?	?	0	0	1	1
Tubiluchus_australensis	1	1	1	1	0	-	-	1	0	1	0	2	1	1	1	0	0	0	?	?	?	?	?	0	0	1	1
Tubiluchus_corallicola	1	1	1	1	0	-	-	1	0	1	0	2	1	1	0	0	1	0	?	?	?	?	?	0	0	1	1
Tubiluchus_philippinensis	1	1	1	1	0	-	-	1	0	1	0	2	1	1	1	1	1	0	?	?	?	?	?	0	0	1	1
Tubiluchus_remanei	1	1	1	1	0	-	-	1	0	1	0	2	1	1	0	1	0	0	?	?	?	?	?	0	0	1	1
Tubiluchus_troglodytes	1	1	1	1	0	-	-	1	0	1	0	2	1	1	0	0	0	0	?	?	?	?	?	0	0	1	1
Tubiluchus_vanuatensis	1	1	1	1	0	-	-	1	0	1	0	2	1	1	0	0	0	0	?	?	?	?	?	0	0	1	1
Tylotites_petiolaris	?	?	?	?	?	0	-	?	?	?	?	?	?	?	?	?	?	?	?	?	?	?	?	?	?	1	1
Xiaoheiqingella_peculiaris	?	?	?	?	?	0	-	?	?	?	?	?	?	?	?	?	?	?	?	?	?	?	?	?	?	1	1
Xystoscolex_boreogyrus	?	?	?	?	?	0	-	?	?	?	?	?	?	?	?	?	?	?	?	?	?	?	?	?	?	1	1
Yunnanpriapulus_halteroformis	?	?	?	?	?	0	-	?	?	?	?	?	?	?	?	?	?	?	?	?	?	?	?	?	?	1	1

Supplementary Table S3 | Dat

	109	110	111	112	113	114
Eokinorhynchus_rarus	2	?	?	0	1	?
Eopriapulites_sphinx	?	1	?	0	1	?
Gastrotricha	0	0	0	1	1	?
Antygomonas	2	0	0	(01)	1	0
Campyloderes	2	0	0	(01)	1	0
Cateria	2	0	0	(01)	1	0
Centroderes	2	0	0	(01)	1	0
Cephalorhyncha	2	0	0	(01)	1	0
Condyloderes	2	0	0	(01)	1	0
Dracoderes	2	0	0	(01)	1	0
Echinoderes	2	0	0	(01)	1	0
Fissuroderes	2	0	0	(01)	1	0
Kinorhynchus	2	0	0	(01)	1	0
Meristoderes	2	0	0	(01)	1	0
Neocentrophyes	2	0	0	(01)	1	0
Paracentrophyes	2	0	0	(01)	1	0
Polacanthoderes	2	0	0	(01)	1	0
Pycnophyes	2	0	0	(01)	1	0
Semnoderes	2	0	0	(01)	1	0
Sphenoderes	2	0	0	(01)	1	0
Triodontoderes	2	0	0	(01)	1	0
Tubulideres	2	0	0	(01)	1	0
Wollunquaderes	2	0	0	(01)	1	0
Zelinkaderes	2	0	0	(01)	1	0
Loricifera	2	0	0	1	1	0
Nematomorpha	2	0	0	0	0	?
Nematoda	2	0	0	(01)	0	?
Aysheaia	0	?	?	?	?	?
Peripatus	0	0	0	1	1	?
Kerygmachela	0	?	?	?	1	?
Microdictyon	0	?	?	?	?	?
Tardigrada	0	0	0	1	0	?
Acanthopriapulus_horridus	3	1	1	0	1	1
Ancalagon_minor	2	?	?	?	?	?
Anningvermis_multispinosus	2	?	?	?	?	?
Chalazoscolex_pharkus	2	?	?	?	1	?
Corynetis_brevis	2	?	?	?	?	?
Cricocosmia_jinningensis	2	?	?	?	?	?
Fieldia_lanceolata	2	?	?	?	?	?
Guanduscolex_minor	2	?	?	?	?	?
Halicryptus_higginsi	1	1	1	0	1	1
Halicryptus_spinulosus	2	1	1	0	1	1
Laojieella_thecata	2	?	?	?	?	?
Louisella_pedunculata	2	?	?	?	?	?
Maccabeus_cirratus	2	1	1	0	1	0
Maccabeus_tentaculatus	2	1	1	0	1	0
Maotianshania_cylindrica	2	?	?	?	?	?
Markuelia	2	?	?	?	?	?
Meiopriapulus_fijiensis	2	1	1	0	1	0
Ottoia_prolifica	2	1	?	?	?	?
Palaeopriapulite_sparvus	3	1	?	?	?	?
Palaeoscolex_piscatorum	2	?	?	?	?	?
Paraselkirkia_jinningensis	2	?	?	?	?	?
Paratubiluchus_bicaudatus	3	?	?	?	?	?
Priapulites_konecniorum	3	1	?	?	?	?
Priapulopsis_australis	3	1	1	0	1	1
Priapulopsis_bicaudatus	3	1	1	0	1	1
Priapulopsis_cnidephorus	2	1	1	?	1	?
Priapulus_abyssorum	3	1	1	0	1	1
Priapulus_caudatus	3	1	1	0	1	1
Priapulus_tuberculatospinosus	3	1	1	0	1	1
Scolecofurca_rara	2	?	?	?	?	?
Selkirkia_columbia	2	?	?	?	?	?
Sicyophorus_rarus	3	1	?	?	?	?
Tabelliscolex_hexagonus	2	?	?	?	?	?
Tabelliscolex_maanshanensis	2	?	?	?	?	?
Tubiluchus_arcticus	2	1	1	0	1	0
Tubiluchus_australensis	2	1	1	0	1	0
Tubiluchus_corallicola	2	1	1	0	1	0
Tubiluchus_philippinensis	2	1	1	0	1	0
Tubiluchus_remanei	2	1	1	0	1	0
Tubiluchus_troglodytes	2	1	1	0	1	0
Tubiluchus_vanuatensis	2	1	1	0	1	0
Tylotites_petiolaris	2	?	?	?	?	?
Xiaoheiqingella_peculiaris	2	?	?	?	?	?
Xystoscolex_boreogyrus	2	?	?	?	1	?
Yunnanpriapulus_halteroformis	2	?	?	?	?	?
